# Microbial rhodoquinone biosynthesis proceeds via an atypical RquA-catalyzed amino transfer from *S*-adenosyl-*L*-methionine to ubiquinone

**DOI:** 10.1038/s42004-022-00711-6

**Published:** 2022-08-01

**Authors:** Trilok Neupane, Lydia R. Chambers, Alexander J. Godfrey, Melina M. Monlux, Evan J. Jacobs, Sophia Whitworth, Jamie E. Spawn, Seo Hee K. Clingman, Kathleen L. Vergunst, Fair M. Niven, James J. Townley, Iris W. Orion, Carly R. Goodspeed, Kathryn A. Cooper, Jeff D. Cronk, Jennifer N. Shepherd, David N. Langelaan

**Affiliations:** 1 Department of Biochemistry & Molecular Biology, Dalhousie University, Halifax, NS Canada; 2 Department of Chemistry and Biochemistry, Gonzaga University, Spokane, WA USA

**Keywords:** Enzymes, Enzyme mechanisms, Biosynthesis

## Abstract

Rhodoquinone (RQ) is a close analogue of ubiquinone (UQ) that confers diverse bacterial and eukaryotic taxa the ability to utilize fumarate as an electron acceptor in hypoxic conditions. The RquA protein, identified in a *Rhodospirillum rubrum* RQ-deficient mutant, has been shown to be required for RQ biosynthesis in bacteria. In this report, we demonstrate that RquA, homologous to SAM-dependent methyltransferases, is necessary and sufficient to catalyze RQ biosynthesis from UQ in vitro. Remarkably, we show that RquA uses SAM as the amino group donor in a substitution reaction that converts UQ to RQ. In contrast to known aminotransferases, RquA does not use pyridoxal 5’-phosphate (PLP) as a coenzyme, but requires the presence of Mn^2+^ as a cofactor. As these findings reveal, RquA provides an example of a non-canonical SAM-dependent enzyme that does not catalyze methyl transfer, instead it uses SAM in an atypical amino transfer mechanism.

## Abstract (web summary)

The RquA protein is required for rhodoquinone biosynthesis; however, its exact function is not yet known. Here, the authors demonstrate that RquA is homologous to SAM-dependent methyltransferases but functions as an aminotransferase using Mn^2+^ as cofactor to convert ubiquinone to rhodoquinone.

## Introduction

**Fig. 1:**
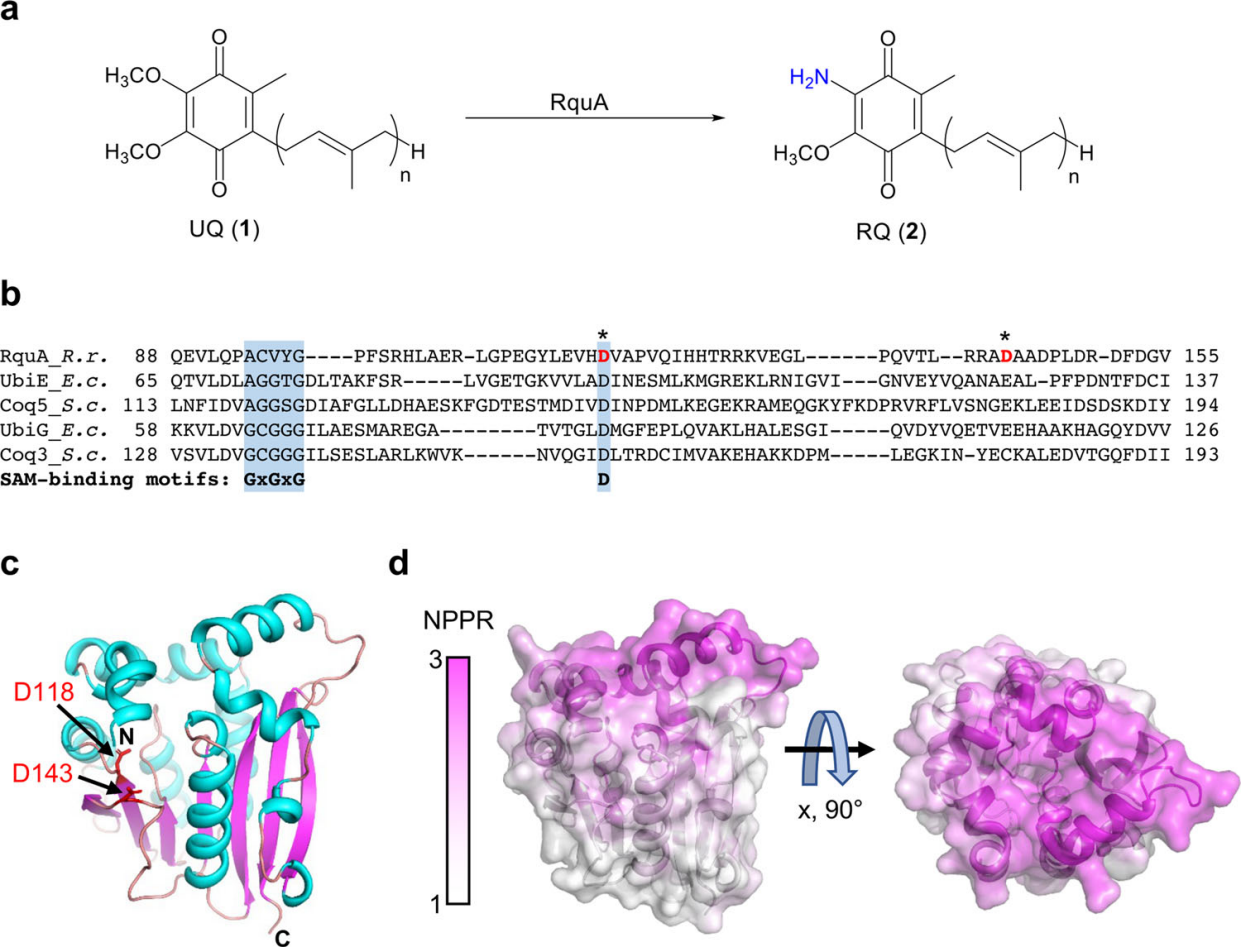
Biosynthesis of rhodoquinone and a structural model of RquA. **a** In select bacteria and protists, RquA converts ubiquinone (UQ, **1**) to rhodoquinone (RQ, **2**). **b** The amino acid sequence of RquA was aligned with related methyltransferases. Sequences of RquA from *R. rubrum* (*R.r*.); UbiE and UbiG from *E. coli* (*E.c*.); and, Coq3 and Coq5 from *S. cerevisiae* (*S.c*.) were aligned using Clustal Omega^63^. Characteristic *S*-adenosyl-*L*-methionine (SAM) binding motifs^21^ are indicated by light blue shading. D118 and D143 of RquA, which participate in interactions with SAM in related methyltransferases, are colored red and indicated with *. **c** An RquA model was predicted by AlphaFold2^64^ using a Google co-laboratory webserver^65^ and visualized as a cartoon using PyMol (Schrödinger, LLC). The first 38 residues, which are not converged or predicted with high confidence by AlphaFold2, are omitted for clarity. α-Helices and β-sheets are colored cyan and magenta, respectively. Aspartic acid residues, D118 and D143, which potentially bind SAM, are colored red. **d** A surface representation is shown of the RquA model colored by a nonpolar-polar ratio (NPPR) as calculated using the Protein-Sol webserver^66^. A large hydrophobic patch (magenta) is present near the putative SAM-binding site.

Quinones play a key role in the bioenergetics of organisms that live in both aerobic and anaerobic environments. Under aerobic conditions, ubiquinone (UQ, **1**, Fig. 1a), a lipid-soluble quinone, accepts electrons from diverse substrates such as NADH and succinate (at complex I and complex II, respectively), reducing it to ubiquinol (UQH_2_). UQ is then regenerated by the multistep transfer of electrons from UQH_2_ to a final electron acceptor, which is often oxygen^1,2^. Other quinones, such as menaquinone and rhodoquinone (RQ, **2**, Fig. 1a), are found in organisms living in anoxic environments^3^. RQ is a benzoquinone that differs only by the substitution of an amino group for one of the methoxy groups on UQ. This results in RQ/rhodoquinol (RQH_2_) having a substantially lower standard redox potential than UQ/UQH_2_ (−63 vs. +110 mV)^4^. The lower redox potential drives complex II in reverse as a fumarate reductase that couples fumarate reduction with the recycling of RQH_2_ to RQ. Thus, when oxygen is limited, the presence of RQ allows organisms to use fumarate as a terminal electron acceptor so that NAD^+^/NADH balance is maintained and ATP production is sustained through a proton electrochemical gradient^5^.

Studies aimed at understanding RQ biosynthesis have determined that there are two distinct biosynthetic pathways. While the phylogenetic distribution of UQ is diverse, RQ is found only in some bacteria, protists, and a few animals, including nematodes, flatworms, molluscs, and annelids^6–9^. Key steps of RQ biosynthesis in animals were recently elucidated^10^. The amino source for RQ biosynthesis in animals is tryptophan, which is metabolized to 3-hydroxyanthranilate through the kynurenine pathway^11,12^ and then polyprenylated by a dedicated COQ-2 isoform. This isoform derives from *coq-2* alternate splicing of an RQ-specific exon, present only in RQ-utilizing animals, while the canonical COQ-2 isoform polyprenylates *para*-hydroxybenzoic acid for UQ biosynthesis.

In contrast to animals, bacteria and protists do not use an alternate isoform of COQ-2 to produce RQ. RQ was initially identified in *Rhodospirillum rubrum*^13^, and in vivo feeding experiments identified UQ as a required substrate for RQ biosynthesis^14^. Comparing the genomic sequences of the F11 strain of *R. rubrum*, which does not produce RQ^15^, and a spontaneous revertant, determined that *rquA*, a putative methyltransferase, is essential for RQ production^16^. Phylogenetic analysis determined that RQ co-occurs with *rquA* in diverse bacteria and that protist *rquA* was gained by lateral gene transfer from bacteria^17^. Expression of recombinant RquA in *Escherichia coli* and yeast resulted in the production of RQ_8_ and RQ_6_, respectively (where RQ_n_ represents the number of isoprenyl units in the tail)^18^. A bioinformatics and transcriptome analysis was unable to identify any other genes in *R. rubrum* that specifically influence RQ biosynthesis^19^, and phylogenetic analysis was unable to identify genes that are genetically linked to *rquA*^17^. These findings collectively suggest that RquA alone is responsible for the conversion of UQ to RQ.

Based on sequence homology, RquA is classified as a class I *S*-adenosyl-*L*-methionine (SAM or AdoMet)-dependent methyltransferase^16^. A structural model of RquA suggests that the SAM-binding site is intact, with conservation of Asp118, which interacts with the O2′ and O3′ of SAM in the related methyltransferase Coq5^20^ (Fig. 1b, c). Asp143 of RquA is also near the SAM-binding site, and the corresponding amino acid in Coq5 interacts with N6 of SAM. Other canonical SAM-binding motifs are disrupted in RquA^16,21^, which suggests it may not function as a methyltransferase. Indeed, non-methylating SAM-dependent enzymes carry out a range of reactions, including decarboxylation, cyclization, and a diverse range of radical-based reactions^21^. Without any well-characterized proteins that have high sequence identity to RquA, or an in vitro assay, the cofactors required for RquA function and the role that SAM plays in RQ biosynthesis have been unclear.

In this report, we isolate RquA and develop an in vitro assay to measure its activity. We identify that RquA catalyzes the conversion of UQ to RQ and requires only Mn^2+^ as a cofactor and SAM as an amino source. This provides an initial characterization of an enzyme that catalyzes a rare amino transfer from SAM and is essential for RQ production to support anaerobic respiration in bacteria and protists.

## Results

### Key aspartate residues of RquA are required for RQ biosynthesis in vivo

To investigate key structural requirements for RquA function, RquA variants were generated and tested for activity in vivo using *E. coli*. The first ~40 amino acids in the RquA sequence do not align well with homologous proteins, and the structure of the N-terminus is not predicted with high confidence by AlphaFold2 due to the absence of homologous sequences (Fig. 1c). Therefore, a truncated Δ40RquA variant was prepared to determine if the N-terminal amino acids are required for folding or activity of RquA. Transformation of BL-21 (DE3) *E. coli* with pET302_RquA or pET302_Δ40RquA resulted in an indistinguishable composition of quinones and indicates that the N-terminal 40 amino acids of RquA are not required for activity (Supplementary Fig. 1a). Two aspartic acid residues, D118 and D143, are predicted to be located in a putative SAM-binding pocket of RquA, and the corresponding amino acids in related methyltransferases (e.g., yeast Coq5) directly interact with SAM^20^. Expression of RquA(D118A/D143A) with both aspartic acid residues replaced with alanine abolished RQ_8_ synthesis, which suggests that SAM binding is essential for RQ_8_ synthesis by RquA (Supplementary Fig. 1b). Given that the N-terminus of RquA is not necessary for function, we assessed if incorporation of an N-terminal fusion protein may enhance the solubility of RquA. Transfection of the XJb (DE3) autolysis strain of *E. coli* with pET21_MBP-RquA resulted in RQ_8_ comprising ~85% of extracted quinones, compared to only ~34% with pET302_RquA (Supplementary Fig. 1a), while non-transformed cells only produced UQ_8_ (Supplementary Fig. 1a, b).

### Expression and purification of RquA

To further investigate RquA function, in vitro assays of RquA activity were developed. Expression of RquA fused to MBP, followed by RquA purification, resulted in optimal protein yield and solubility. MBP-RquA (73.1 kDa) was purified by amylose affinity chromatography. Overnight TEV protease cleavage followed by nickel affinity chromatography allowed for a crude separation of RquA (28.9 kDa) from MBP (43.3 kDa). Final purification by size exclusion chromatography resulted in high-purity RquA, which has a measured mass within 1 Da of its calculated molecular weight as determined by ESI mass spectrometry (Supplementary Fig. 2). RquA elutes from a calibrated size exclusion column earlier than MBP, with a radius of hydration that corresponds to an estimated molecular mass of ~81 kDa. This suggests that RquA assembles in solution as a homodimer bound to detergent. An additional minor peak at ~50 mL corresponded to high molecular mass contaminating proteins and high-order oligomers of RquA and MBP-RquA.

To assess if purified RquA was correctly folded, a fluorescence spectroscopy-based in vitro assay was used to determine if purified RquA was capable of binding to SAM (Supplementary Fig. 3). RquA has several tryptophan residues and measurable intrinsic fluorescence. Upon titration of RquA with SAM, a modest reduction of fluorescence intensity was observed. The change in fluorescence intensity at 340 nm was plotted and fit to a one-site binding model to determine that RquA binds SAM with a dissociation constant (*K*_d_) of 2221 ± 1019 µM. The error with the measured K_d_ between RquA and SAM is quite large, which is in part due to the inability to saturate the interaction due to its modest affinity for SAM in absence of UQ.

### A balanced redox environment and divalent metal cations are required for RquA activity

An in vitro assay was developed to determine required buffer additives and cofactors that support RquA activity. The assay was performed using SAM, and a synthetic UQ_3_ substrate and production of RQ_3_ was measured using liquid chromatography-mass spectrometry (LC-MS). Several buffers were found to be suitable for supporting RquA activity in vitro, ranging from pH 6 to 8, with TRIS (pH 8) providing the highest and most reproducible yields of RQ_3_ (Supplementary Fig. 4a). To better stabilize the protein and hydrophobic quinone substrate, glycerol (10%) and Brij-35 detergent (0.05%) were added to the assay which significantly increased activity (Supplementary Fig. 4c). It was determined that a low concentration of reducing agent is required for RquA activity, and tris(2-carboxyethyl)phosphine (TCEP, 0.5 mM) was found to be superior to dithiothreitol (DTT) and glutathione (GSH) for this purpose (Supplementary Fig. 5a). RquA was unable to catalyze the conversion of UQ to RQ under anoxic conditions (Supplementary Fig. 5c), but activity could be rescued by the addition of oxygenated reaction buffer. A metal screen was performed by treating purified MBP-RquA with EDTA, which eliminated all activity (Supplementary Fig. 6a). Introduction of nine different divalent metal cations in our assay revealed that Mn^2+^ significantly facilitated RquA (500 pmol) activity, allowing ~82% conversion of UQ_3_ (1000 pmol) to RQ_3_ in 32 min (817 ± 64 pmol RQ_3_). Both Co^2+^ and Fe^2+^ provided modest activity, with 11% (112 ± 21 pmol RQ_3_) and 5% (50 ± 2 pmol RQ_3_) conversion of UQ_3_ to RQ_3_, respectively (Supplementary Fig. 6a). Minimal production of RQ_3_ (12 ± 0.5 pmol RQ_3_) was also achieved in the presence of Zn^2+^ and DTT (2.5 mM) (Supplementary Fig. 5b).

### Additional cofactors or alternate substrates do not support RquA activity

Additional in vitro assays indicated that MBP-RquA and TEV-cleaved RquA had a similar activity with assays containing Mn^2+^, while MBP alone gave no activity (Supplementary Fig. 4d). Therefore, these RquA proteins were used interchangeably in subsequent assays. The addition of pyridoxal 5′-phosphate (PLP), a known cofactor in transamination reactions, did not enhance the activity of MBP-RquA or RquA (Supplementary Figs. 4c, 6c, respectively). Further, purified RquA has no absorbance maxima at 420 nm that can be attributed to PLP conjugation^22^. RquA retains activity when solubilized from inclusion bodies under denaturing conditions and refolded, suggesting that non-covalent cofactors are not required for RQ biosynthesis (Supplementary Fig. 7). Removal of SAM from the MBP-RquA assay eliminated the production of RQ_3_, and addition of the SAM analogs *S*-adenosyl-*L*-homocysteine (SAH) or sinefungin failed to restore activity (Supplementary Fig. 6b).

Alternate quinone substrates for RquA were investigated to further probe its mechanism of action. A small amount of RQ_3_ (26 ± 22 pmol/500 pmol protein) was detected when UQ_3_H_2_ was used as a substrate instead of UQ_3_; however, a no protein control revealed that UQ_3_H_2_ becomes ~10% air oxidized to UQ_3_ over the course of the assay, which likely reacted with RquA (Supplementary Fig. 8b). Assays performed using potential intermediates demethylubiquinone (DMeQ_3_) or its hydroquinone (DMeQ_3_H_2_) showed that neither support RQ_3_ production (Supplementary Fig. 8b).

### Kinetics of MBP-RquA

**Fig. 2:**
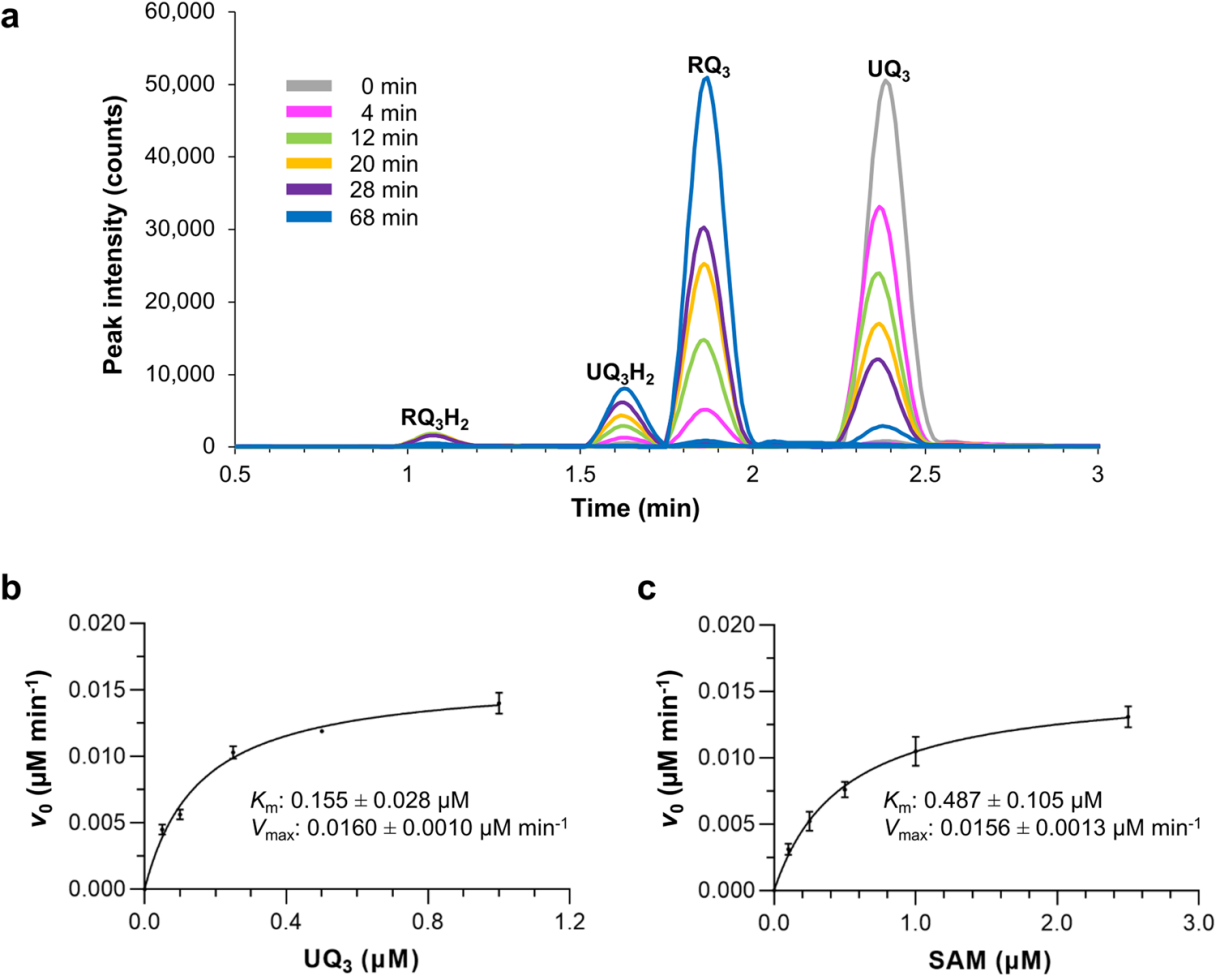
RquA kinetic study. **a** Time course chromatograms measuring the production of rhodoquinone-3 (RQ_3_) are overlaid for an MBP-RquA in vitro assay monitored by liquid chromatography-mass spectrometry (gray: 0 min, magenta: 4 min, green: 12 min, orange: 20 min, purple: 28 min, blue: 68 min) after ubiquinone-3 (UQ_3_) (1 μM) was added to MBP-RquA (0.5 μM) with *S*-adenosyl-*L*-methionine (SAM, 5 μM). **b** A Michaelis–Menten curve was generated for varying UQ_3_ concentrations (0.05–1 µM) at 0, 4, 8, and 16 min time points with MBP-RquA (0.5 μM) and SAM (5 μM). **c** A second Michaelis–Menten curve was generated for varying SAM concentrations (0.1–2.5 µM) at the same time points with MBP-RquA (0.5 μM) and UQ_3_ (5 μM). The *V*_max_ corresponds to a peak specific activity of 0.22 pmol min^−1^ μg protein^−1^. For **b**, **c**, *n* = 3 independent experiments and error bars represent the standard deviation of the mean.

A time course chromatogram acquired in real-time during an MBP-RquA in vitro assay shows the immediate production of RQ_3_H_2_ and RQ_3_ with peak retention times of 1.07 and 1.86 min, respectively, and the disappearance of UQ_3_ at 2.38 min (Fig. 2a). Over time, the RQ_3_H_2_ peak disappeared, and a UQ_3_H_2_ peak at 1.63 min increased in intensity. After 68 min, the reaction slowed and there was little change in product peak areas (chromatograms at 124 min not shown). The initial velocities of MBP-RquA (0.5 µM) with varying concentrations of UQ_3_ (0.05–1 µM) were measured with a saturating concentration of SAM (5 µM). The data were fitted to Michaelis–Menten kinetics to determine an apparent *K*_m_ of 0.155 ± 0.028 µM and *V*_max_ of 0.0160 ± 0.0010 µM min^−1^ for UQ_3_ (Fig. 2b). The same experiment was performed with SAM at concentrations ranging from 0.1–2.5 µM with saturating UQ_3_ (5 µM). The apparent *K*_m_ for SAM was nearly three times that of UQ_3_ at 0.487 ± 0.105 µM and the *V*_max_ was similar at 0.0156 ± 0.0013 µM min^−1^ (Fig. 2c). Based on this data, the *k*_cat_ for RquA was calculated to be 0.031 ± 0.001 min^−1^, or roughly a turnover of two molecules RQ_3_/molecule RquA/h. The measured affinity of RquA for SAM is weaker than the corresponding *K*_m_ value. This may indicate that RquA interacts with SAM and UQ_3_ in a cooperative manner since the binding assay was completed in the presence of only SAM, while the kinetic assays contained both SAM and UQ_3_.

### SAM is the amino donor for RQ biosynthesis in vivo and in vitro

**Fig. 3:**
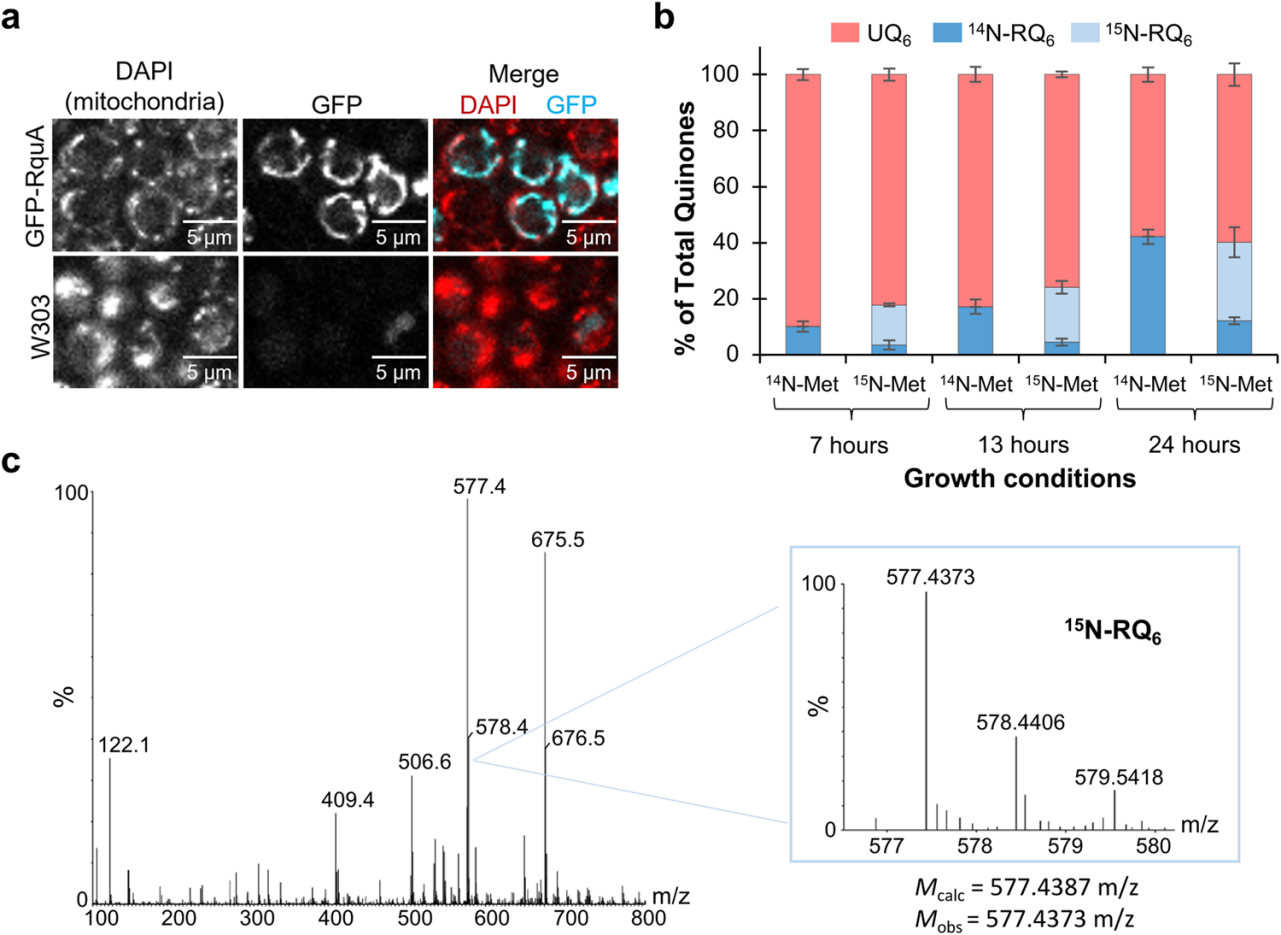
Expression of GFP-RquA in *S. cerevisiae*. **a** Confocal microscopy images of yeast expressing GFP-RquA were imaged at 405 nm (DAPI: red) and 510 nm (GFP: cyan). The merged image (top right) shows an overlap of the GFP with the DAPI, indicating the localization of RquA in the mitochondria. **b** Yeast harboring pRCM_GFPRquA produce rhodoquinone-6 (RQ_6_, dark blue), and supplementation with ^15^N-*L*-methionine yields ^15^N-RQ_6_ (light blue), The production of ubiquinone-6 (UQ_6_) is shown in red. *n* = 2 biologically independent samples and error bars represent the standard deviation of the mean. **c** The experimental mass of synthesized [^15^N-RQ_6_ + H]^+^ was determined to be 577.4373 *m/z* (C_38_H_58_^15^NO_3_ exact mass = 577.4387 *m/z*, mass accuracy = −2.4 ppm).

To investigate whether SAM is the amino donor for RQ biosynthesis in vivo, a green fluorescent protein (GFP)-tagged RquA was expressed in W303 *Saccharomyces cerevisiae*, which was previously shown to produce RQ_6_ with heterologous expression of RquA^18^. Successful production of GFP-RquA was confirmed using confocal microscopy and the protein was found to be localized in the mitochondria using DAPI staining of mitochondrial DNA (Fig. 3a)^23^. Since SAM is biosynthesized from *L*-methionine and ATP by SAM synthetase (SAMS)^24^, yeast expressing GFP-RquA were grown in SD-Ura media supplemented with either ^14^N-*L*-methionine or ^15^N-*L*-methionine and cultures were harvested at varying time points. Lipid extracts were analyzed for the presence of ^14^N-RQ_6_, ^15^N-RQ_6_, and UQ_6_. Cultures supplemented with ^15^N-Met produced predominantly ^15^N-RQ_6_, though some ^14^N-RQ_6_ was also produced (Fig. 3b). The proportion of ^14^N-RQ_6_ increased over time, which may be a result of isotope scrambling due to protein degradation^25^. The identity of ^15^N-RQ_6_ was confirmed with high mass accuracy (−2.4 ppm) using time-of-flight mass spectrometry (Fig. 3c).

**Fig. 4:**
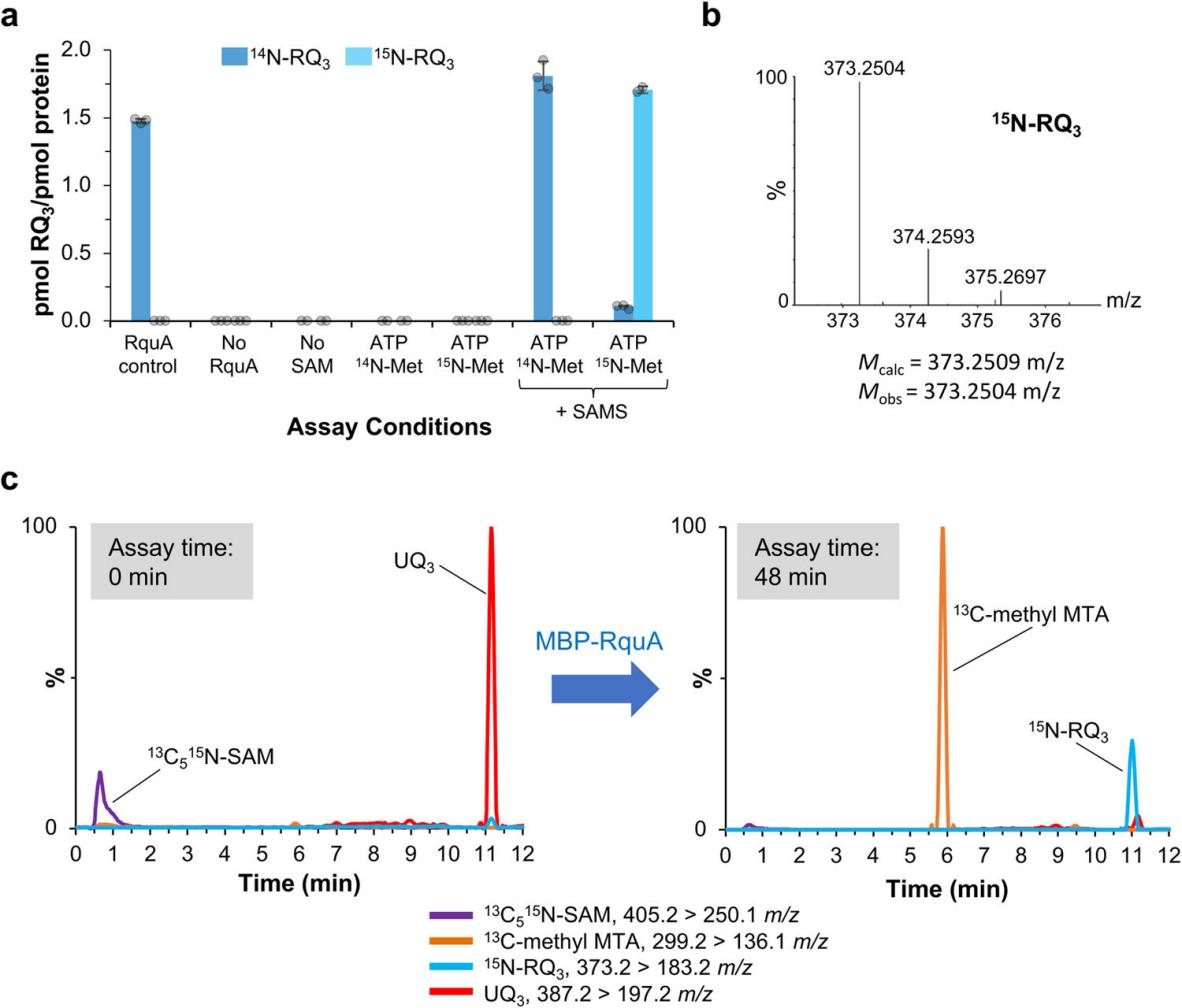
SAM is the amino donor for RQ biosynthesis. **a** In vitro assays were performed with commercially available *S*-adenosyl-*L*-methionine (SAM, 5 μM) or a SAM synthetase (SAMS) reaction mixture containing ATP and ^14^N-*L*-methionine or ^15^N-*L*-methionine. Cleaved RquA (0.5 μM) and ubiquinone-3 (UQ_3_, 5 μM) were used with assay conditions described previously. Production of ^14^N-rhodoquinone-3 (^14^N-RQ_3_) and ^15^N-RQ_3_ are shown as dark and light blue, respectively. *n* = 3 independent experiments and error bars represent the standard deviation of the mean. **b** The mass of synthesized [^15^N-RQ_3_ + H]^+^ was determined to be 373.2504 *m/z* (C_23_H_34_^15^NO_3_ exact mass = 373.2509 *m/z*, mass accuracy = −1.4 ppm). The full mass spectrum is presented in Supplementary Fig. 11. **c** Purified ^13^C_5_^15^N-SAM (5 μM), MBP-RquA (0.5 μM), and UQ_3_ (5 μM) were used in an assay at pH 6. Four multiple reaction monitoring transitions (^13^C_5_^15^N-SAM: purple, ^13^C-methylthioadenosine (MTA): orange, ^15^N-RQ_3_: blue, UQ_3_: red) were monitored during the assay as outlined above. The response factor for MTA is approximately four times greater than RQ_3_ and 100 times greater than SAM under these chromatography conditions. Peak intensities were normalized out of 100%, based on the largest peak in each chromatogram.

As a supplementary approach to verify that the amino group from SAM is used directly in RQ biosynthesis, we prepared both ^14^N-SAM and ^15^N-SAM in vitro using purified SAMS and ATP with either ^14^N-Met or ^15^N-Met substrates^26^. The SAMS reaction products were added directly into a RquA in vitro assay and production of ^14^N-RQ_3_ or ^15^N-RQ_3_, respectively, was observed (Fig. 4a). The identity of ^15^N-RQ_3_ was confirmed using time-of-flight mass spectrometry with a mass accuracy of −1.4 ppm (Fig. 4b). Omission of RquA, SAM, or SAMS from the assay resulted in no RQ_3_. The commercially available ^15^N-Met contains up to 4% of the ^14^N isotope, and therefore a minor amount of ^14^N-RQ_3_ was also observed in the ^15^N-Met assay. Finally, free ammonia cannot be incorporated into RQ by RquA in vitro. When the reaction was carried out in the presence of ^15^NH_4_^+^, with or without SAM, no ^15^N-RQ_3_ was produced (Supplementary Fig. 8a).

### MTA, methanol, bicarbonate, and an aldehyde hydrate are products of the RquA reaction

The in vitro assay was further used to identify 5′-methylthioadenosine (MTA) as an additional product of RquA that is derived from SAM. We used SAMS, ATP, and either ^14^N-Met or U^13^C_5_^15^N-Met to synthesize SAM and ^13^C_5_^15^N-SAM, respectively. These SAM isotopes were purified and included in the RquA in vitro assay. LC-MS analysis of the reaction mixture identified MTA (and ^14^N-RQ_3_) or ^13^C-methyl MTA (and ^15^N-RQ_3_) as reaction products of RquA when SAM or ^13^C_5_^15^N-SAM, respectively, are used as substrates (Supplementary Figs. 4c, 9a). This indicates that the methyl group in SAM is not donated during the RquA reaction since the ^13^C-methyl from ^13^C_5_^15^N-SAM remains bonded to sulfur in MTA. MTA can be produced by non-enzymatic degradation of SAM; however, significantly more MTA was detected in the presence of RquA (average of 8.45x more), indicating that the observed MTA is due to RquA activity (Supplementary Fig. 9b).

**Fig. 5:**
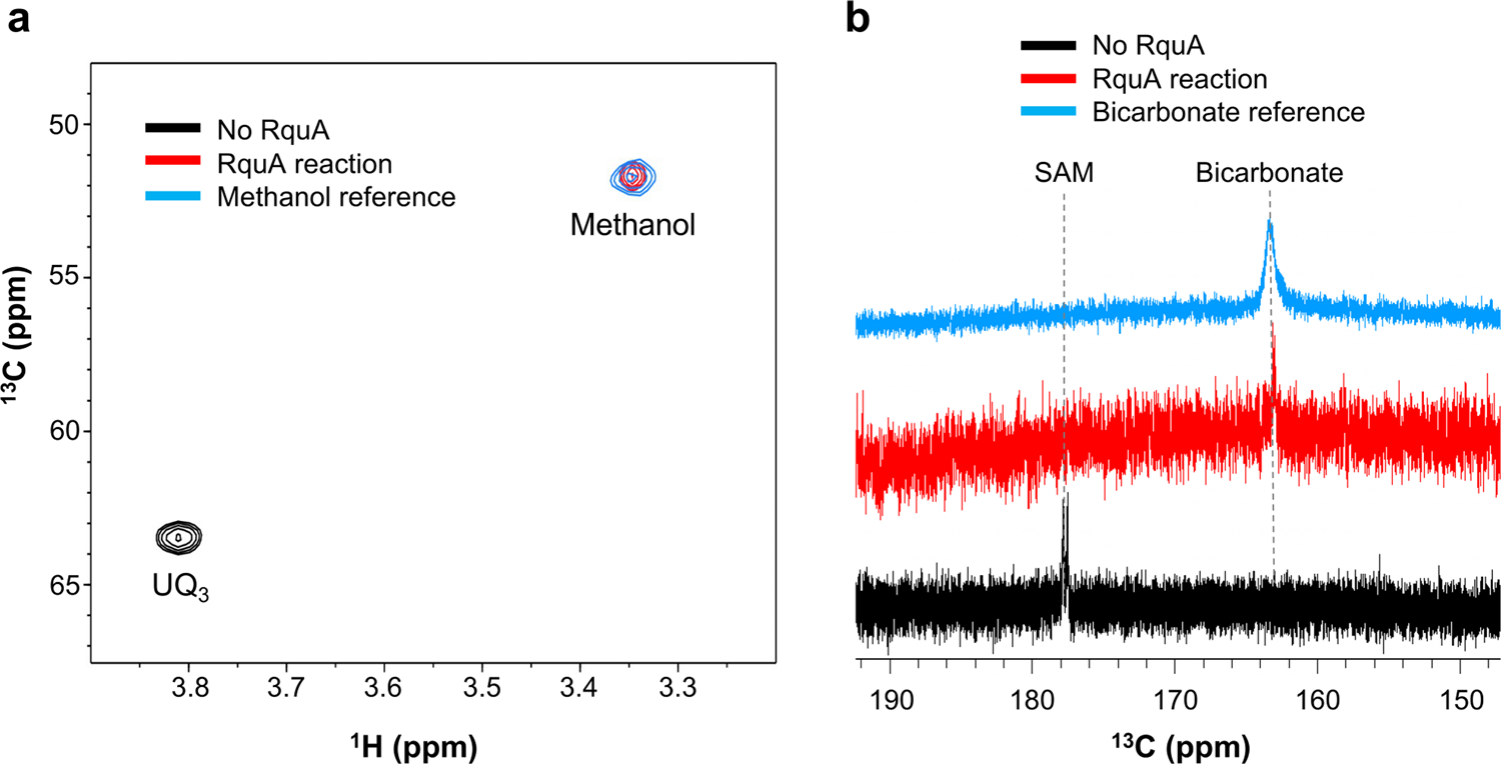
Identification of RquA reaction products by NMR. **a** ^13^C-methanol is a product generated by RquA from ubiquinone-3 (UQ_3_). Assays were performed using purified *S*-adenosyl-*L*-methionine (SAM, 400 µM), 5-^13^C-methoxy-UQ_3_ (200 µM), and RquA (25 µM) in assay buffer. ^1^H-^13^C spectra are overlaid for a control sample lacking RquA (black), the complete reaction (red), and for a sample of 0.1% methanol in assay buffer (blue). **b** Bicarbonate is detected in the RquA in vitro reaction. Assays were performed using purified ^13^C_5_^15^N-SAM (200 µM), UQ_3_ (350 µM), and RquA (25 µM) in assay buffer. Stacked ^1^H-decoupled ^13^C spectra are shown for a control sample lacking RquA (black), for the complete reaction (red), and for a sample of sodium bicarbonate (50 mM) in assay buffer (blue).

NMR spectroscopy was used to identify additional products of the RquA reaction. Using a UQ_3_ substrate with a ^13^C-labeled 5-methoxy group for the reaction, ^1^H-^13^C HSQC experiments detected the appearance of a product with chemical shifts that overlap with methanol (Fig. 5a). Additional SAM-derived products were detected by carrying out the in vitro reaction with ^13^C_5_^15^N-SAM. A ^1^H-decoupled ^13^C spectrum of the reaction mixture indicated that bicarbonate is produced during the reaction, which could form from dissolved CO_2_ and is consistent with the elimination of CO_2_ from SAM (Fig. 5b). Finally, ^1^H-^13^C HSQC, as well as HSQC-TOCSY experiments, detected the production of MTA and a ^13^C-labeled aldehyde hydrate containing at least three carbon atoms, which is identifiable by a characteristic ^13^C chemical shift of ~93 ppm and correlations between the aliphatic carbons that are observed in HSQC-TOCSY experiments (Supplementary Fig. 10). This hydrate is likely derived from an aldehyde generated on the methionine portion of SAM following decarboxylation/deamination and MTA cleavage.

## Discussion

RquA is essential for the biosynthesis of RQ in *R. rubrum*^16^, and when expressed in *E. coli* or *S. cerevisiae*, recombinant RquA converts UQ to RQ^18^. The phylogenetic analysis determined that RquA has not co-evolved with any other proteins^17^, and RNAseq and bioinformatic screens were unable to identify other genes that are essential for RQ production in *R. rubrum*^19^. These studies suggest that RquA is solely responsible for the conversion of UQ to RQ; however, there have been no published in vitro studies of RquA that definitively show this.

In this work, we developed an in vitro assay using purified RquA to determine which factors are essential for its activity. Detergents such as Brij-35 were required for RquA solubility during purification and for in vitro assays, and the presence of glycerol also enhanced RquA activity (Supplementary Fig. 4c). Detergent micelles can mimic a lipid membrane^27^, and their requirement for RquA solubility along with a putative hydrophobic patch (Fig. 1d) suggest that RquA is a membrane-bound monotopic protein. RquA requires specific divalent metal ions, with Mn^2+^ allowing for the most activity while low activity was observed in the presence of Co^2+^ and Fe^2+^. Mn^2+^ and Mg^2+^ have similar coordination chemistry and can often be interchanged if they only play a structural role in a protein^28^. The strict requirement of Mn^2+^ over Mg^2+^ suggests that Mn^2+^ is directly involved in catalysis and does not simply play a structural role in RquA. A balanced redox environment appears to be important, with both aerobic conditions and a reducing agent being required for RquA function. While oxygen was required for the in vitro reaction, likely another oxidant is utilized in vivo since the RquA protein is native to *R. rubrum* which is capable of photosynthetic and fermentative growth in the absence of oxygen^29^. A reducing agent may be needed either to maintain the stability of RquA or to reduce reaction intermediates. Low activity was also achieved with Zn^2+^ in the presence of excess thiol reducing agent. Although zinc is not redox active on its own, it has been shown to facilitate redox chemistry when coordinated with sulfur^30^.

**Fig. 6:**
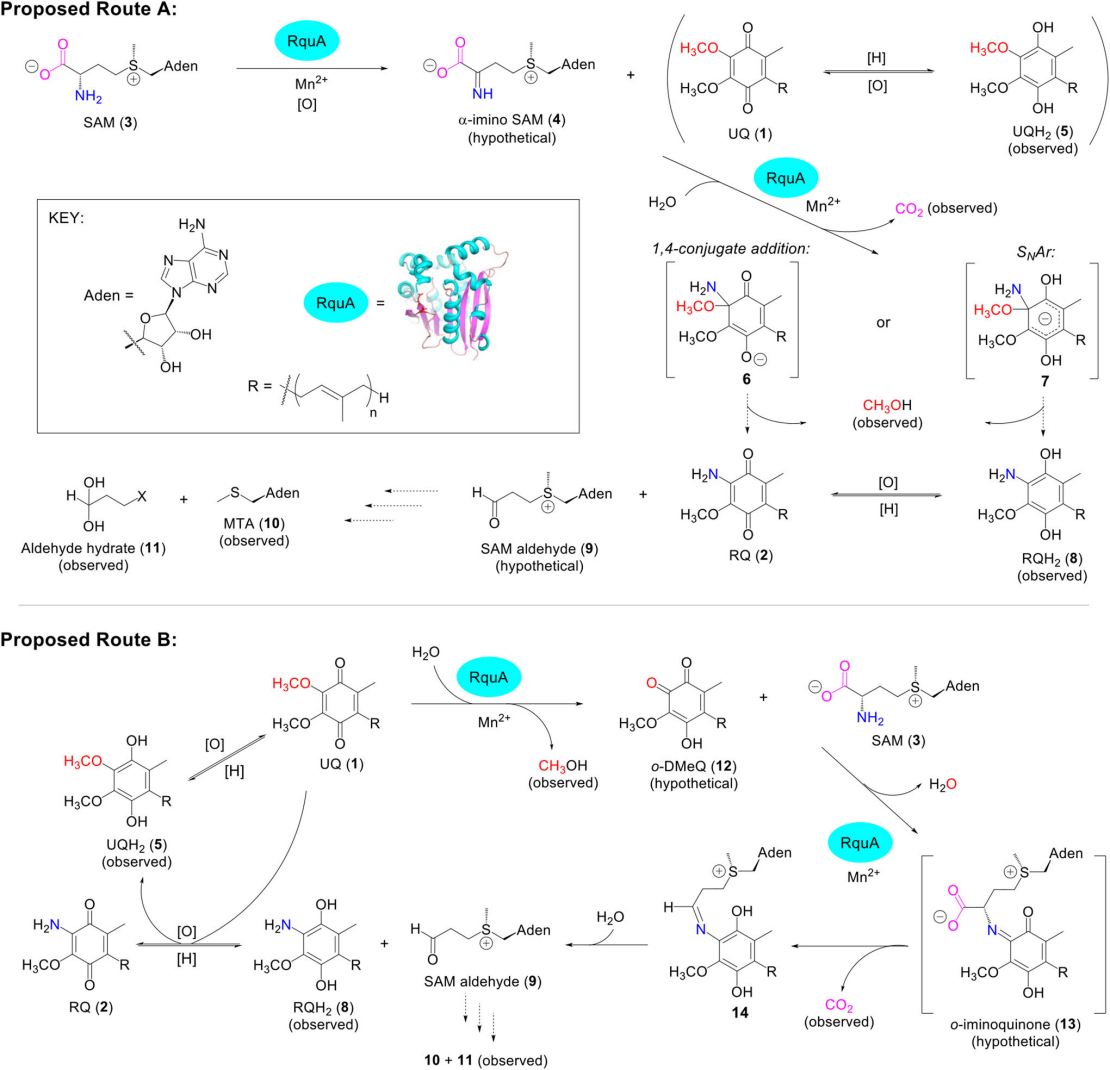
Proposed routes for the RquA reaction. Ubiquinone (UQ, **1**) and *S*-adenosyl-*L*-methionine (SAM, **3**) are required substrates of RquA for the biosynthesis of rhodoquinone (RQ, **2**). The α-amino group (blue) from SAM is transferred to form RQ and decarboxylation (magenta) is observed. The reaction requires the presence of Mn^2+^ and both ubiquinol (UQH_2_, **5**) and rhodoquinol (RQH_2_, **8**) intermediates are detected during the in vitro assay, as well as CH_3_OH (red), methylthioadenosine (MTA, **10**), and an aldehyde hydrate (**11**). Proposed Route A involves oxidation to form α–imino SAM (**4**), followed by decarboxylation and amino substitution via either a 1,4-conjugation addition (intermediate **6**) or a nucleophilic aromatic substitution (S_N_Ar, intermediate **7**) to produce RQ or RQH_2_, respectively. Proposed Route B first assumes the demethylation of UQ to form *ortho*-demethylubiquinone (*o*-DMeQ, **12**) before the addition of SAM to form an *o*-iminoquinone (**13**), which generates RQH_2_ after decarboxylation and hydrolysis. RQH_2_ can be oxidized to RQ in either route. Both routes also propose the formation of a SAM-aldehyde product, which through reaction with a nucleophile (X), can decompose to MTA and the observed aldehyde hydrate.

Several potential mechanisms have been proposed to describe the conversion of UQ to RQ by RquA^16,18,19^. RquA has non-canonical SAM-binding motifs that suggest it may not carry out a typical methyltransferase reaction despite having a methyltransferase fold^16^. This was the basis of two recent proposals for RquA catalysis^6^: (1) RquA may act as an amidotransferase that uses SAM as an electrostatic catalyst and, through 1,4-conjugate, addition-elimination replaces the methoxy group of UQ with an amine derived from an amino donor such as glutamine^6^; or (2) RquA catalyzes the demethylation of UQH_2_ to produce demethylubiquinol (DMeQH_2_) which can be oxidized to an orthoquinone for transamination, using RquA as a PLP-dependent aminotransferase to transfer the amino group from SAM to create RQ. Surprisingly, these proposed mechanisms are inconsistent with our in vitro assay results as they involve either an additional amino source or the use of PLP as a cofactor, neither of which are necessary for RquA function. Refolded RquA retains function, intact mass analysis indicates RquA does not have any covalently bound cofactors, and PLP does not enhance the reaction rate of RquA, all of which suggest that RquA does not require any additional cofactors to convert UQ to RQ. We also only observe UQ_3_H_2_ or RQ_3_H_2_ as reaction intermediates, not DMeQ_3_/DMeQ_3_H_2_. These in vitro assays indicate that RquA does not require other proteins for function and directly converts UQ to RQ, with only a requirement of SAM, Mn^2+^, and a reducing agent (Fig. 6).

This study identifies RquA as a member of a diverse group of non-canonical SAM-dependent enzymes that do not catalyze methyl transfer. Alternate enzymatic roles for SAM include functions such as cyclization, epoxidation, and decarboxylation (reviewed in refs. ^21,31,32^). To the best of our knowledge, the only other enzyme that catalyzes the transfer of the SAM α-amino group is BioA (formerly DAPA synthase) which transfers the amino group to 7-keto-8-aminopelargonic acid (KAPA) during biotin biosynthesis. However, BioA does not require divalent metal for activity, but instead requires PLP for transamination and is classified as a member of the PLP-dependent aminotransferase group of enzymes^33,34^. In contrast, RquA has homology to SAM-dependent methyltransferases and does not require PLP, suggesting that it follows a different mechanism to achieve amino transfer from SAM. In another approach, some SAM-dependent enzymes use radical chemistry to catalyze diverse reactions^21,31,32^; however, these reactions require homolytic cleavage of the 5′C-S bond of SAM to generate a 5′-deoxyadenosyl radical and use a Fe-S cluster to facilitate electron transfer. We observe that with RquA, the 5′C-S bond of SAM is not cleaved during the reaction because MTA is detected as a product. Also, when ^13^C-methyl SAM is used as a substrate ^13^C-methyl MTA is formed. Further, RquA does not have conserved cysteine residues, making the presence of a Fe-S cluster and radical chemistry involving SAM unlikely. Our finding that RquA catalysis is best supported by divalent metals that can change their oxidation state (e.g., +2 to +3) suggests that RquA catalysis may involve electron transfer. Electron transfer and cycling of Mn^+2^ to Mn^+3^ during catalysis has been observed in other mononuclear Mn^2+^-dependent enzymes such as superoxide dismutase^35^, oxalate oxidase^36^, oxalate decarboxylase^37^, homoprotocatechuate 3,4-dioxygenase^38^, and lipoxygenase^39^ (reviewed in ref. ^40^). Further, Schiff bases and ammonia are known ligands for manganese (II) or (III) complexes^41^ and RquA may stabilize such a complex to aid in the transfer of an ammonia or amide ligand to UQ or UQH_2_.

Our results establish that RQ biosynthesis requires the substitution of a methoxy group on UQ with a SAM-derived amino group, with consequent production of CO_2_, MTA, methanol, and an aldehyde hydrate. We propose two distinct RquA reaction routes that are consistent with these results (Fig. 6). In proposed route A, SAM (**3**) is first oxidized to form α-imino SAM (**4**). Similar oxidation occurs with amino acid oxidases^42,43^ as well as some dehydrogenases^44,45^ that use nucleotide cofactors. Since none of these cofactors are required by RquA, UQ may instead serve as the hydrogen acceptor for this reaction (Fig. 6). It is well-established that UQ can be reduced to semiquinone and hydroquinone species in the respiratory chain^46,47^. After production of α-imino SAM, decarboxylation may then occur along with amino transfer to UQ or UQH_2_ (**5**) via 1,4,-conjugate addition (intermediate **6**) or nucleophilic aromatic substitution (intermediate **7**; S_N_Ar), respectively, with a corresponding loss of methanol. A SAM-aldehyde product (**9**) would be produced, which could then decompose into MTA (**10**) and the observed aldehyde hydrate (**11**) via substitution with a nucleophile (X) to cleave the C-S bond. Alternatively, the SAM-aldehyde product may undergo elimination following a route proposed for 7,8-diaminopelargonic acid aminotransferase^33^ to form MTA and an α,β-unsaturated aldehyde, which could then undergo conjugate addition with a nucleophile (X) to generate the observed aldehyde hydrate.

Reaction route A (Fig. 6) is consistent with a recently described S_N_Ar mechanism^48^. 5-Nitroanthranilic acid aminohydrolase uses enzyme-coordinated M^2+^ to bind the aromatic substrate 5-nitroanthranilic acid and catalyze the substitution of the amino group with a hydroxyl group. This mechanism relies on divalent metals (Mn^2+^ or Zn^2+^) to catalyze the reaction and a nitro substituent to stabilize the intermediate^48^. While there are no strongly electron-withdrawing substituents on UQH_2_, it is possible that coordination of the methoxy substituent on the ring by Mn^2+^ may sufficiently stabilize an anionic intermediate, allowing for the addition of ammonia and elimination of methanol. Interestingly, RQ_3_/RQ_3_H_2_ were not formed in our assay when UQ_3_H_2_ was added directly as a substrate, suggesting that the process of quinone reduction may be coupled to the deamination of SAM. Alternatively, a RquA-manganese complex could facilitate a 1,4-conjugate addition of NH_3_/NH_2_^-^ to the more electrophilic UQ, followed by the elimination of CH_3_OH/CH_3_O^-^. A similar non-enzymatic conjugate addition reaction between UQ_10_ and NH_4_OH has been reported, which generates RQ_10_ and iso-RQ_10_^49^.

An alternate proposed mechanism (Fig. 6, route B) instead begins with an O-demethylation of UQ, possibly facilitated by Mn^2+^, to form methanol and *o*-DMeQ (**12**), which could then react with SAM to form an *o*-iminoquinone (**13**). This intermediate could decarboxylate and hydrolyze to form RQH_2_ (**8**) and the same SAM-aldehyde proposed in route A, which could decompose into MTA and the observed aldehyde hydrate. There are many O-demethylation mechanisms that have been characterized, and this step may involve Mn^2+^ coordination to the oxygen of the 5-methoxy group on UQ in order to activate the methyl group for nucleophilic attack by water^50^. The proposed nucleophilic addition involving the SAM amino group is consistent with other reactions observed in the biosynthesis of amino acid-derived natural products^51^ and the conversion of tryptophan to an aminoquinone^52^.

Both routes A and B involve the decarboxylation of SAM, which may follow any number of mechanisms (reviewed in^53^). In particular, RquA may decarboxylate SAM via mechanisms that do not require a cofactor (e.g., orotidine monophosphate decarboxylase^54^), require Mn^2+^ and oxygen (e.g., oxalate decarboxylase^55^), or simply require a divalent metal cation (e.g., α-amino-β-carboxymuconate-ε-semialdehyde decarboxylase^56^). Both routes A and B are consistent with the observed production of UQH_2_; in route A, reduction of UQ may be coupled with SAM oxidation, and in route B, reducing equivalents from RQH_2_ may be used to generate UQH_2_ from UQ. These redox reactions could occur through direct electron transfer or be mediated by metal ions or a reducing agent.

We have developed the first in vitro assay to monitor the conversion of UQ to RQ by RquA and determined that RquA facilitates a PLP-independent amino transfer from SAM to UQ. Our results indicate that RquA defines a class of non-methylating SAM-dependent enzymes able to carry out Mn-dependent amino transfer reactions. This conversion requires only SAM, Mn^2+^, and a balanced redox environment—no other proteins, cofactors, or amino sources are required. We have suggested some possible mechanisms of RquA catalysis, and further experimental verification of these potential mechanisms is required. Future studies will seek to build an accurate description of RquA catalysis by monitoring the oxidation state of manganese during catalysis, characterizing the structure of RquA with substrates or substrate analogs, and identifying the catalytic steps required to create RQ.

## Methods

### Plasmid construction

#### pET21_MBP-RquA and pET21_RquA(D118A/D143A)

A plasmid was purchased (Bio Basic Inc., Markham, ON) that coded for RquA from *R. rubrum* [UniprotID: Q2RPC3] with an additional 5′ and 3′ BamHI and XhoI recognition sites, respectively. This plasmid was digested with BamHI and XhoI restriction endonucleases and ligated into a modified pET21 vector using T4 DNA ligase. The final plasmid consisted of an open reading frame coding for a hexahistidine tag, maltose binding protein, a tobacco etch virus (TEV) protease cleavage site, and RquA (MBP-RquA). A second plasmid coding for a D118A and D143A variant of RquA was purchased, and the same procedure was used to generate pET21_RquA(D118A/D143A), which codes for an N-terminal hexahistidine tag, TEV protease cleavage site, and RquA.

#### pET302_RquA and pET302_Δ40RquA

The *rquA* gene [Rru_A3227] was amplified by PCR from chromosomal *R. rubrum* DNA using Pfu Ultra II Hotstart Master Mix with primers P1 and P2 (Supplementary Table 1). The *rquA* amplicon and pET302/NT-His vector were separately digested with EcoRI and BamHI, purified, and then ligated using T4 DNA ligase to create a plasmid coding for RquA with an N-terminal hexahistidine tag (pET302_RquA). The truncated *rquA* gene (minus 120 bp on 5′-end; *Δ40rquA*) was amplified by PCR from a pET303_RquA vector template^18^ using Q5^®^ High Fidelity Master Mix with primers P3 and P4 (Supplementary Table 1). The *Δ40rquA* amplicon was digested and ligated into pET302/NT vector as described above, to create pET302_Δ40RquA.

#### pET21_SAMS

The *metK* gene coding for SAM synthetase was amplified by PCR from *E. coli* BL-21 (DE3) competent cells using Q5^®^ High Fidelity Master Mix with primers P5 and P6 (Supplementary Table 1; New England Biolabs). The *metK* amplicon and a modified pET21 vector were separately digested with BglII and XhoI, purified, and then ligated using T4 DNA ligase to create a plasmid coding for SAM synthetase with an N-terminal hexahistidine tag (pET21_SAMS).

#### pRCM_GFP-RquA

The pRCM_GFP-RquA plasmid was constructed using restriction cloning at the KpnI and ClaI cut sites of the pRCM multi-copy vector^57^. The GFP-RquA gene insert (1545 bp) containing a C-terminal hexahistag coding region and KpnI and ClaI restriction sites was synthesized by SynBio.

For this plasmid and the others described above, ligation reactions were used to transform *E. coli* DH5α cells using ampicillin for selection, and plasmids were isolated from single colonies. All plasmid sequences were verified by Sanger sequencing. Primers used for cloning are listed in Supplementary Table 1.

### Native expression and purification of RquA

Chemically competent *E. coli* XJb (DE3) autolysis cells were transformed with pET21_MBP-RquA. Single bacterial colonies were grown at 37 °C in LB media supplemented with ampicillin (100 µg/mL) and arabinose (3 mM). When the cultures reached an optical density at 600 nm (OD_600_) of 0.8–1.0, expression was induced with isopropyl β-d-1-thiogalactopyranoside (IPTG, 0.5 mM). Cells were allowed to grow overnight (18–20 h) at 20 °C and were then harvested by centrifugation. Cell pellets were resuspended in lysis buffer (TRIS (20 mM), NaCl (200 mM), TCEP (1 mM), and 0.1% Brij-35 adjusted to pH 8.0), lysed by a freeze-thaw cycle and sonication, and clarified by centrifugation at 4 °C (25,000 × *g* for 20 min). Lysis supernatant was applied to a gravity column containing amylose resin (New England Biolabs, Ipswich, MA), washed with lysis buffer, and eluted with lysis buffer containing maltose (10 mM). TEV-protease (0.13 mg per liter of cell culture) was added to the eluted protein overnight at 4 °C. The cleaved MBP-RquA was passed through a nickel affinity column (IMAC Sepharose 6 Fast Flow; Cytiva, Marlborough, MA) and the flowthrough fraction containing RquA was collected and further purified using a HiLoad 16/60 Superdex 200 gel filtration column (Cytiva) equilibrated with TRIS (20 mM), NaCl (200 mM), TCEP (1 mM), and 0.1% Brij-35 adjusted to pH 8.0. Fractions containing soluble RquA were pooled and concentrated using a 50 kDa molecular weight cut-off centrifugal filter, flash frozen in liquid nitrogen, and stored at −80 °C for later use. The purification process was confirmed by mass spectrometry and SDS-PAGE with visualization by Coomassie Brilliant Blue R-250. MBP-RquA was isolated following the same procedure as above, except that the cleavage and nickel affinity purification steps were omitted.

### Purification and refolding of RquA from inclusion bodies

RquA was overexpressed using XJb (DE3) autolysis *E. coli* cells harboring the pET303_RquA vector as previously described^18^. Cell pellets (1 g) were lysed by two freeze-thaw cycles and resuspended in 20 mL of lysis buffer containing TRIS (20 mM), NaCl (200 mM), and TCEP (1 mM) adjusted to pH 8. The lysate was treated with DNase I (25 µg/mL) and Problock^TM^ Gold Protease Inhibitor Cocktail. Inclusion bodies were pelleted at 12,000 × *g* for 30 min at 4 °C and washed twice with lysis buffer containing 2 M urea (10 mL). The final pellet was solubilized in lysis buffer with 8 M urea (10 mL). The denatured RquA-His was purified by nickel affinity chromatography using a 5 mL HisTrap FF column (GE Healthcare, Chicago, IL) with an elution buffer containing imidazole (300 mM), urea (8 M), TRIS (20 mM), NaCl (200 mM), TCEP (1 mM) and 0.1% Brij-35. The protein was refolded by overnight dialysis at 4 °C in a Slide-A-Lyzer Cassette (ThermoFisher, Waltman, MA) with two buffer exchanges containing TRIS (20 mM), NaCl (200 mM), MnCl_2_ (0.5 mM), 10% glycerol, and 0.1% Brij-35. As a control for assays with refolded RquA, MBP-cleaved RquA (10 µM) was dialyzed under the same conditions. Both refolded and cleaved RquA were used in assays at 1 µM final concentration.

### SAM synthetase purification

For overexpression BL-21 (DE3) *E. coli* were transformed with pET21_SAMS. Overexpression proceeded as for MBP-RquA, except that after the addition of IPTG, cultured were incubated for 4 h at 37 °C before centrifugation. Cell pellets were resuspended in binding buffer containing TRIS (20 mM), NaCl (200 mM), β-mercaptoethanol (5 mM), adjusted to pH 8.0, lysed, and clarified as described above. The supernatant was loaded onto a nickel affinity column and washed with a binding buffer containing imidazole (20 mM). SAMS was eluted from the resin with a binding buffer containing imidazole (300 mM), and sample purity was assessed by SDS-PAGE (Supplementary Fig. 12).

### Expression of His-RquA and His-Δ40RquA

BL-21 (DE3) *E. coli* were transformed with pET302_RquA or pET302_Δ40RquA. Single transformant colonies were used to inoculate overnight cultures in LB media (5 mL) supplemented with ampicillin (100 µg/mL) at 37 °C. Outgrowth cultures (15 mL) were prepared the next day using 150 µL of overnight inoculum and grown for 2.5 h to an OD_600_ of 0.4. Cultures were cooled to 30 °C before treating with 100 µM IPTG and growth was allowed to proceed for 17 h at 30 °C. Untransformed BL-21 (DE3) cells were grown in parallel without ampicillin. Cultures were harvested by centrifugation of 5-mL aliquots and pellets were frozen at −80 °C until lipid extraction.

### Expression of GFP-RquA in yeast

W303 yeast was transformed with pRCM_GFPRquA using standard protocols with a modified 40 min heat shock, and transformants were selected using SD minus uracil (SD-Ura) media^58^. Overnight cultures grown at 30 °C in SD-Ura were prepared from single colony scrapes and used to inoculate 20-mL cultures for expression of GFP-RquA. Growth was performed at 30 °C in SD-Ura media containing ^14^N-*L*-methionine (0.5 mM) or ^15^N-*L*-methionine (0.5 mM, 96–98%) for 7–24 h. Cells were harvested by centrifugation at 4000 × *g* for 5 min at 4 °C using the following culture volumes per pellet: 7 h (20 mL), 13 h (10 mL), and 24 h (5 mL).

### Fluorescence binding experiments

RquA (10 μM) was prepared in assay buffer contain TRIS (20 mM), NaCl (200 mM), TCEP (1 mM), 0.05% Anapoe-35, and 5% methanol, adjusted to pH 8.0. RquA samples were titrated with SAM and intrinsic tryptophan fluorescence was measured from 300 to 450 nm with an excitation at 290 nm, in an interval of 1 nm (Cary Eclipse fluorescence spectrophotometer; 5 nm excitation/emission slit widths). Changes in tryptophan fluorescence intensity at 340 nm upon addition of SAM were fit to a single binding site model and used to determine the dissociation constant (*K*_d_) of the interaction.

### In vitro RquA assay general protocols

Triplicate assays to screen metals, protein variants, and required cofactors were performed in volumes of 1 mL. Assays were conducted using TRIS buffer (35 mM), NaCl (100 mM), TCEP (0.5 mM), 10% glycerol, and 0.05% Brij-35 adjusted to pH 8. Except for the EDTA metal rescue assays, all assay mixtures contained 0.5 mM MnCl_2_. Protein samples (MBP-RquA, RquA, or MBP) were first diluted to 1 µM in assay buffer and then added as a 0.5 mL aliquot to assays. Mixtures were allowed to equilibrate for 5 min before the addition of quinone substrate. For the metal rescue assays, EDTA (0.5 mM) was added to the protein mixture and allowed to chelate for 5 min before adding M^2+^ salts at a concentration of 1 mM (M^2+^ salts used were: MnCl_2_·4H_2_O, MgCl_2_·6H_2_O, ZnSO_4_·7H_2_O, FeCl_2_·4H_2_O, CaCl_2_·2H_2_O, Cu(NO_3_)_2_·H_2_O Cd(NO_3_)_2_·4H_2_O, or Ni(NO_3_)_2_·6H_2_O). Following the preincubation period, UQ_3_ (10 µL of a 100 µM stock in EtOH, 1 µM final) was added to initiate the reaction. Reactions were allowed to proceed for 32 min with shaking at rt and were quenched by direct addition of the mixtures to EtOH (1.5 mL). A UQ_4_ internal standard (200 pmol) was added before mixtures were extracted twice with hexanes (2 × 2 mL). The combined organic extracts were dried with nitrogen gas and resuspended in EtOH (1 mL) for LC-MS analysis. Time course chromatograms were acquired at rt by injecting 20 µL samples at designated time intervals from LC-MS vials containing 1 mL assay mixtures without quenching or extraction.

### Buffer optimization assays

Six buffers were tested for use in the in vitro assays. TRIS, phosphate, and bicarbonate buffers were prepared at 35 mM final concentration and adjusted to pH 8 using TRIS base, KH_2_PO_4_, or NaHCO_3_, respectively. Assays were performed with MBP-RquA as described in the general protocols. Some precipitation of Mn_3_(PO_4_)_2_ occurred in the phosphate buffer upon the addition of MnCl_2_. Buffer pH was evaluated using additional buffers prepared at 35 mM final concentrations: CHES (pH 9), TRIS (pH 7), and MES (pH 6). Assays were performed alongside the TRIS (pH 8) buffer as previously described. However, after 32 min at rt, these assays were quenched with HCl (10 µL, 5 M) and UQ_4_ standard (200 pmol) was added prior to LC-MS analysis (no extractions were performed). Additional optimization assays were performed following general protocols using TRIS (pH 8) with the omission of glycerol, Brij-35, or MnCl_2_, as well as with the addition of PLP (1 µM). The use of PLP as a cofactor was further explored with cleaved RquA (0.5 µM) in time course assays with or without PLP (5 µM). Assays were quenched with acid at 4, 8, 16, and 32 min and analyzed as described above. All assays were performed in triplicate.

### Reducing agent assays

Cleaved RquA (10 µM) was dialyzed overnight at 4 °C in buffer containing either TCEP (1 mM), DTT (1 mM), glutathione (GSH,1 mM), or no reducing agent. Each of these dialysis buffers contained TRIS (20 mM), NaCl (200 mM), Brij-35 (0.1%), and glycerol (10%) adjusted to pH 8. The dialyzed proteins were diluted in assays to a final concentration of 0.5 µM in buffer supplemented with MnCl_2_ (0.5 mM) and other buffer components described in the general protocols. An assay with no reducing agent was compared to others containing TCEP (0.5 mM final), DTT (0.5 mM final), GSH (0.5 mM final), oxidized glutathione (GSSG, 0.5 mM final), or a GSH:GSSG mixture (both at 0.25 mM final). A second experiment was performed using MBP-RquA diluted to 0.5 µM in buffer containing 2.5 mM DTT and the other components listed above; however, the protein was treated with EDTA (0.5 mM) for 5 min before the addition of either Mn^2+^ or Zn^2+^ (1 mM MnCl_2_ or ZnSO_4_). All assays contained SAM (5 µM) and were initiated by the addition of UQ_3_ (1 µM). Assays were performed in triplicate and quenched with acid after 32 min at rt and analyzed directly using LC-MS with a UQ_4_ internal standard.

### Anaerobic assays

Solutions of RquA (1 µM) and a no protein control (TRIS buffer with 0.1 mM TCEP), and 2X concentrated assay buffer were prepared in 10-mL Schlenk flasks. Prior to deoxygenation, aliquots were removed for aerobic assays that were conducted simultaneously. Oxygen was removed from the solutions by slowly sparging the assay buffer and RquA solutions with argon for 45 min. SAM and UQ_3_ solutions were sparged with argon for 15 min. Anaerobic assays (1 mL) were assembled in 5-mL round bottom flasks containing septa and stir bars and kept under argon. The 50% anaerobic assays (anaerobic buffer + aerobic RquA) were performed under argon in LC-MS vials with septa, and the 50% aerobic assays (aerobic buffer + anaerobic RquA) and aerobic assays were assembled in microcentrifuge tubes as previously described. The assay volume contained 0.5 mL RquA or no protein buffer solutions, which yielded final assay concentrations of 35 mM TRIS, 10% glycerol, 0.05 mM TCEP, 0.5 mM Mn^2+^, 1 µM UQ_3_, and 5 µM SAM. Anaerobic assay components were added by air-tight syringes and needles were first flushed with argon. Anaerobic solutions and assays remained under an argon atmosphere throughout assembly and incubation. All assays were quenched with acid after 32 min, followed by the addition of the UQ_4_ internal standard (200 pmol), and samples were diluted 1:10 in EtOH prior to analysis by LC-MS.

### Alternate substrate assays

Alternate substrates were tested in vitro using cleaved RquA (0.5 µM) and compared with a UQ_3_ control (1 µM) using the general assay conditions previously described. A reduced UQ_3_H_2_ substrate (46 µM) was prepared from UQ_3_ in EtOH using a few crystals of NaBH_4_ and added directly to the assay (22 µL, 1 µM final) after gas evolution ceased. Demethylubiquinone-3 (DMeQ_3_) was synthesized as previously reported^59^ and reduced to DMeQ_3_H_2_ (35 µM) as described above. Oxidized or reduced forms were added directly to assays (29 µL, 1 µM final). Controls using substrates without protein were analyzed at 0 and 32 min to determine whether any redox reactions occurred in the buffer. All assays were quenched with acid and analyzed directly using LC-MS with a UQ_4_ internal standard.

### Kinetics assays

All kinetics experiments were performed with MBP-RquA (0.5 µM) using single pot reactions with aliquots removed at designated time points (4, 8, 16, 32, and 64 min). A 25-mL round bottom flask with a magnetic stir bar was used for the reaction vessel with a total assay mixture volume of 7 mL. The same buffer components were used as described in the previous section. For assays that varied in UQ_3_ (0.05–1 µM), SAM was added at 5 µM. For assays that varied in SAM (0.1–2.5 µM), UQ_3_ was added at 5 µM. Timed aliquots (0.5 mL) were removed and added to EtOH (0.75 mL) to quench the reaction. UQ_4_ standard (100 pmol) was added to the ethanolic mixtures prior to extraction with hexanes (2 × 1 mL). Extracts were dried and resuspended in EtOH (0.5 mL) for analysis. Experiments were repeated in triplicate. Initial velocities were determined from plots of pmol RQ_3_ produced over time (min), using the slopes generated from 0–16 min time points. Michaelis–Menten plots were prepared using GraphPad Prism 9.2 software, and a nonlinear fit was performed to determine apparent *K*_m_ and *V*_max_.

### SAM isotope assays and MTA analysis

Additional SAM derivatives were prepared from ^13^C-labeled *L*-methionine isotopes (^13^C-methyl and U^13^C_5_^15^N, Cambridge Isotopes Laboratories, Tewksbury, MA) using methods previously described in ref. ^26^. The SAMS reaction mixtures were purified by ion exchange chromatography using a 1 mL HiTrap SP column (GE Healthcare, Chicago, IL) in sodium acetate buffer (20 mM, pH 4.3) with an elution buffer containing NaCl (1 M) following the step gradient method described by ref. ^60^. Unlabeled SAM (5 µM), ^13^C-methyl SAM (5 µM), or ^13^C_5_^15^N-SAM (5 µM) were used in assays with MBP-RquA in MES buffer (35 mM) adjusted to pH 6 to better stabilize SAM and MTA products. Reaction mixtures were sampled (20 µL) and analyzed directly by LC-MS after 48 min without quenching. For quantitation, assays were quenched with acid and UQ_1_ (400 pmol) and UQ_4_ (200 pmol) internal standards were added. Chromatography was performed using a C18 column (Luna C18(2) 3 µm, 100 Å, 50 × 2 mm, Phenomenex, Torrance, CA) at a flow rate of 0.5 mL/min using the same instruments and buffers described in the LC-MS quantitation section. The gradient (buffer A-buffer B) method used was: 0 to 3 min (100:0), 3 to 6 min (100:0 to 70:30), 6 to 7.5 min (70:30), 7.5 to 8.5 min (70:30 to 5:95), 8.5 to 12.5 min (5:95), 12.5 to 13.5 min (5:95 to 100:0), 13.5 to 15 min (100:0)^61^. SAM isotopes were eluted at 0.6 min, MTA isotopes were eluted at 5.8 min, UQ_1_ eluted at 9.5 min, farnesylated quinones (^14^N-RQ_3_, ^15^N-RQ_3_, and UQ_3_) eluted between 11–11.2 min, and UQ_4_ eluted at 11.8 min. Ions were monitored using MRM transitions of singly charged ions from each analyte precursor ion ([M + H]+) to its respective product ion ([M]+) with instrument and software parameters reported previously^18^. Additional analyte-specific parameters are listed in Supplementary Table 2.

### SAM synthetase assays

Assays were performed in triplicate with commercially available SAM (5 µM, NEB, Ipswich, MA) or 5 µL of a SAMS reaction mixture containing: SAMS (17.4 µM), TRIS (50 mM), KCl (100 mM), MgCl_2_ (20 mM), ATP (1 mM), and ^14^N-*L*-methionine (5 mM) or ^15^N-*L*-methionine (5 mM, 96–98%, Cambridge Isotopes Laboratories, Tewksbury, MA) adjusted to pH 8^26^. The SAMS reaction was allowed to proceed 2 h at 30 °C before aliquots were added to the RquA assay. Cleaved RquA (0.5 µM) and UQ_3_ (5 µM) were used with assay conditions and extraction methods described previously. To obtain high-resolution data, the assay concentrations were scaled up 10X for analysis using time-of-flight mass spectrometry.

### Synthesis of 5-^13^C-methoxy-UQ_3_

DMeQ_3_ (31 mg, 0.084 mmol) was dissolved in extra dry acetone (3 mL, Fisher Scientific, Hampton, NH) and anhydrous K_2_CO_3_ (58 mg, 0.042 mmol) was added to the mixture, followed by a crystal of 18-crown-6 (Sigma-Aldrich, Saint Louis, MO). After the solution turned dark brownish purple, ^13^CH_3_I (52 µL, 0.84 mmol, Cambridge Isotopes Laboratories, Tewksbury, MA) was added dropwise and the reaction was heated at reflux for 4 h. The reaction mixture turned orange upon completion and was cooled and filtered through celite prior to purification by silica gel column (1.5 × 12 cm, 9.5:0.5 hexanes:ethyl acetate), yielding 5-^13^C-methoxy-UQ_3_ as an orange oil (16 mg, 50%). TLC: *R*_*f*_ 0.6 (9:1 hexanes:ethyl acetate). ^1^H NMR (CDCl_3_, 400 MHz) δ: 1.57 (s, 3H), 1.58 (s, 3H), 1.66 (s, 3H), 1.73 (s, 3H), 1.97 (m, 8H), 1.99 (s, 3H), 3.18 (d, 2H, *J* = 7.0 Hz), 3.97 (d, 3H, *J* = 164 Hz), 3.99 (s, 3H), 4.93 (m, 1H), 5.06 (m, 2H); ^13^C NMR (CDCl_3_, 100 MHz) δ: 11.97, 12.41, 16.03, 16.34, 17.69, 25.32, 25.72, 26.46, 26.74, 39.71, 61.18*, 61.40, 118.88, 123.86, 124.31, 131.33, 135.21, 137.61, 138.88, 141.69, 144.35, 183.94, 184.79. *^13^C-labeled methoxy carbon; HRMS (*m/z*): [M + H]^+^ calcd for C_23_^13^CH_34_O_4_, 388.2569; found, 388.2553. Spectra are shown in Supplementary Figs. 13, 14.

### NMR in vitro assay

All samples prepared for NMR experiments contained sodium phosphate (20 mM), NaCl (50 mM), MnCl_2_ (0.05 mM), TCEP (0.5 mM), 0.1% Brij-35, and DSS (500 µM) adjusted to pH 8. The in vitro assay was carried out in sample buffer with RquA (25 µM) and was incubated at 28 °C for 4 h. Assays also contained (i) 5-^13^C-methoxy-UQ_3_ (200 µM) and SAM (400 µM) for the detection of ^13^C-methanol or (ii) UQ_3_ (350 µM) and ^13^C_5_^15^N-SAM (200 µM) for the detection of other SAM-derived products. Additional control samples consisted of the in vitro assay lacking RquA, or of sample buffer containing either 0.1% methanol, 20 mM MTA, or 50 mM sodium bicarbonate. For detection of SAM-derived products, after reactions were complete, the assay was filtered through a 10 kDa spin concentrator, which served to remove signals from UQ_3_, RQ_3_, detergent, and RquA, which are all micelle associated and unable to pass through the membrane pores. ^1^H-decoupled ^13^C, ^1^H-^13^C HSQC, and ^1^H-^13^C HSQC-TOCSY experiments of each sample were collected at 25 °C using a 500 MHz Bruker Avance Spectrometer equipped with a room temperature BBFO SmartProbe or 700 MHz Bruker Avance III Spectrometer equipped with a TXI cryoprobe (NMR-3 and NRC-IMB facilities, respectively). Peak assignments were determined by overlaying spectra of RquA assay with the control reactions.

### Lipid extraction of cell pellets and standards for LC-MS analysis

Quinones were extracted from cell pellets using previously described protocols and volumes were scaled according to pellet size^18^. Unless noted otherwise, all cell extracts were resuspended in a final volume of 1 mL for LC-MS analysis. For BL-21 *E. coli* strains, ~20 OD_600_ units were pelleted in triplicate and UQ_6_ internal standard (2500 pmol) was added prior to extraction. For XJb *E. coli* strains, ~5 OD_600_ units were pelleted in duplicate. For pET21_MBP-RquA samples, 1000 pmol of UQ_6_ standard was added prior to extraction, and for pET21_RquA(D118A/D143A) samples, 250 pmol of UQ_6_ standard was added. A calibration curve was prepared for *E. coli* quinone quantitation using extracted standards containing UQ_6_ (5 pmol/20 µL injection), RQ_9_ (0.5, 1.5, 3.0, 6.0, or 12 pmol/20 µL injection) and UQ_9_ (0.5, 1.5, 3.0, 6.0, or 12 pmol/20 µL injection). RQ_9_ and UQ_9_ were used for quantitation since RQ_8_/UQ_8_ standards were not available. *S. cerevisiae* samples were collected in duplicate. Pellets collected after 7 h contained ~2 OD_600_ units and 100 pmol of UQ_3_ internal standard was added prior to extraction. The 7 h extracts were resuspended in a final volume of 100 µL for LC-MS. Pellets collected at 13 and 24 h contained ~24 OD_600_ units per pellet and 1000 pmol of UQ_3_ was added before extraction. For yeast quantitation, extracted standards contained UQ_3_ (20 pmol/20 µL injection) and UQ_6_ (0.6, 1.2, 2.4, 6.0, or 12.0 pmol/20 µL injection). Since an RQ_6_ standard was not available, the quantity of RQ_6_ was determined using a pmol conversion from the UQ_6_ standard curve and applying an RQ/Q response correction factor of 2.45^18^. For in vitro assays, a calibration curve was prepared using standards treated in assay buffer for the same length of time as assays, and then extracted or acid quenched following the assay protocol. Standards used for in vitro assay analysis on a Waters Quattro Micro TQ contained UQ_4_ (4 pmol/20 µL injection), RQ_3_ (0.2, 0.8, 4, 8, 16, and 32 pmol/20 µL injection) and UQ_3_ (0.4, 1.6, 8, 16, 32, and 64 pmol/20 µL injection). UQ_1_ (0.4 pmol/5 µL injection) was used as an internal standard for MTA quantitation (0.025, 0.05, 0.125, 0.25, and 0.5 pmol MTA/5 µL injection) and concentrations of quinone standards were diluted tenfold for analysis on a Xevo TQ-S Cronos.

### LC-MS quantitation and high-resolution mass determination

Lipid extracts from cell pellets, in vitro assay extracts, and standards were separated using high-performance liquid chromatography (Waters Alliance 2795 or Acquity UPLC, Waters Corporation, Milford, MA), and quinones were quantified using a triple quadrupole mass spectrometer in positive electrospray mode (Waters Micromass Quattro Micro or Xevo TQ-S Cronos, Waters Corporation, Milford, MA). Chromatography was performed with the sample changer maintained at 12 °C for extracted samples, or 21 °C for real-time assays, using a Luna PFP[2] column (50 × 2 mm, 3 µm, 100 Å, Phenomenex, Torrance, CA) at a flow rate of 0.5 mL/min and injection volumes of 20 µL (Quattro Micro) or 5 µL (Xevo Cronos). For quinones extracted from cell pellets, a 9-min gradient method was used as previously described^18^. For in vitro assays, quinones were eluted between 1 and 4.5 min by using a gradient system containing water with 0.1% formic acid (buffer A) and acetonitrile with 0.1% formic acid (buffer B). The water and acetonitrile used were LC-MS-grade Optima (Fisher Scientific, Pittsburgh, PA), and the formic acid was >99% packaged in sealed 1-mL ampoules (Thermo-Scientific, Rockford, IL). The gradient (buffer A:buffer B) method used was as follows: 0 to 3.5 min (30:70), 3.50 to 3.75 min (30:70 to 2:98), 3.75 to 4.75 min (2:98), 4.75 to 5 min (2:98 to 30:70), and 5 to 6.5 min (30:70). Quantitation was accomplished using multiple reaction monitoring (MRM) with QuanLynx or TargetLynx data processing software^18^. Additional quinone-specific parameters not defined previously are listed in Supplementary Table 3^18^. The quinone pmol were determined from the standard curves and the amount of internal standard added. In vivo samples were normalized by OD_600_ units pelleted, and percentages of UQ and RQ were determined out of the total pmol of quinones. For yeast, ^15^N-RQ_6_ peak areas were corrected to remove counts contributed by other M + 1 peak areas found naturally in ^14^N-RQ_6_ (namely, ^13^C-RQ_6_). High-resolution mass determination of ^15^N-RQ_6_, ^15^N-RQ_3_, ^15^N-SAM, and 5-^13^C-methoxy-UQ_3_ was performed using an LCT Premier XE time-of-flight mass spectrometer (Waters Corporation, Milford, MA) in V-positive electrospray mode using a leucine-enkephalin reference and separation of quinones was achieved using a Waters Acquity UPLC (Waters Corporation, Milford, MA) with the same chromatography conditions above.

### Confocal microscopy

Confocal microscopy was performed with live yeast cells from both W303 and W303 transformed with pRCM_GFP-RquA, grown on SD-complete and SD-Ura, respectively. Colonies from agar plates were swabbed and resuspended in 1 mL of respective media. DAPI (1 μM) was added to each sample to stain the mitochondrial DNA^23^ and the cells were incubated at 30 °C for 10 min. Squares (1 × 1 cm) of SD-complete and SD-Ura agar were cut and placed on top of microscope slides^62^. After the incubation period, 10 μL of the yeast cultures were added to the respective agar squares and a coverslip was placed on top for immobilization. The cells were then imaged using a Leica TCS-SPEII confocal microscope with an ACS APO 63x/1.3 N.A. objective with LAS X software. The DAPI images were recorded at a wavelength of 405 nm, and the GFP images were recorded at a wavelength of 510 nm. The images were processed using FIJI to increase brightness and contrast.

### Reporting summary

Further information on research design is available in the Nature Research Reporting Summary linked to this article.

## Supplementary information

Supplementary Information

Reporting Summary

## Data Availability

The data that support the findings of this study are available from the corresponding authors upon reasonable request.
