# Supplementary material for: Microbial rhodoquinone biosynthesis proceeds via an atypical RquA-catalyzed amino transfer from S-adenosyl-L-methionine to ubiquinone
Source: Commun Chem. 2022 Aug 1;5:89. doi: 10.1038/s42004-022-00711-6 (PMC9814641; doi:10.1038/s42004-022-00711-6)
Supplement: Supplementary file 1 — Supplementary Information [file 42004_2022_711_MOESM1_ESM.pdf]

SUPPLEMENTARY INFORMATION FOR:

Microbial rhodoquinone biosynthesis proceeds via an atypical RquA-catalyzed amino transfer from *S*-adenosyl-*L*-methionine to ubiquinone

Trilok Neupane[1], Lydia R. Chambers[2], Alexander J. Godfrey[2], Melina M. Monlux[2], Evan J. Jacobs[2], Sophia Whitworth[2], Jamie E. Spawn[2], Seo Hee K. Clingman[2], Kathleen L. Vergunst[1], Fair M. Niven[2], James J. Townley[2], Iris W. Orion[2], Carly R. Goodspeed[2], Kathryn A. Cooper[2], Jeff D. Cronk[2], Jennifer N. Shepherd[2*] and David N. Langelaan[1*]

[1]Department of Biochemistry & Molecular Biology, Dalhousie University, Halifax, NS, Canada
[2]Department of Chemistry and Biochemistry, Gonzaga University, Spokane WA, USA
[*]Co-corresponding authors: shepherd@gonzaga.edu and david.langelaan@dal.ca

Contents:

Supplementary Figures 1-15

Supplementary Tables 1-3

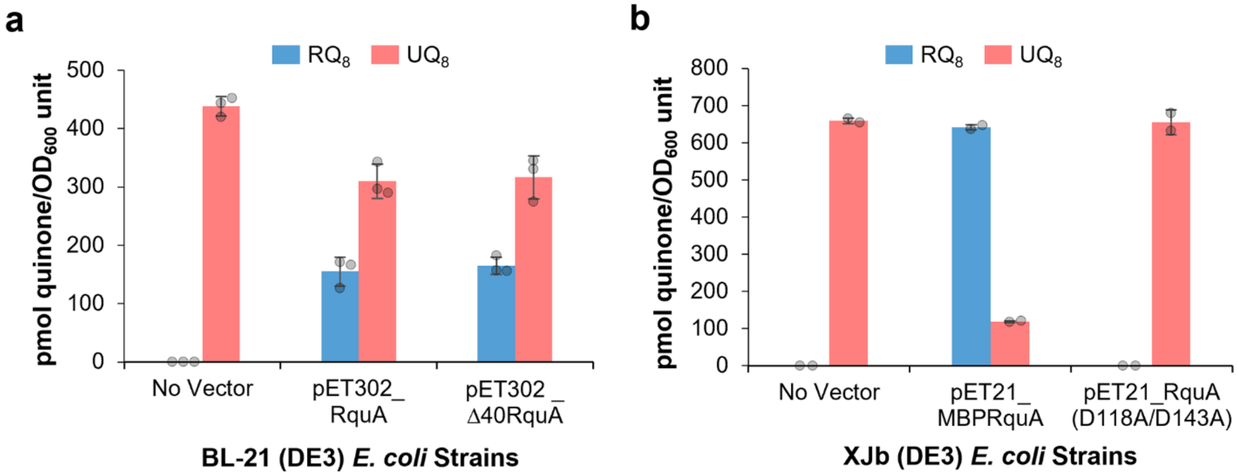

Supplementary Figure 1. Quinones produced *in vivo* in *E. coli* grown with and without RquA expression vectors. a BL-21 (DE3) *E. coli* were transformed with plasmids containing either the full *rquA* gene or a truncated sequence missing the first 120 bp (Δ40RquA). **b** XJb (DE3) *E. coli* were transformed with plasmids for expression of maltose binding protein (MBP)-tagged RquA or a mutant with the D118 and D143 codons changed to A118 and A143. After growth, cell pellets were collected and the percentage of ubiquinone-8 (UQ_8, red) and rhodoquinone-8 (RQ_8, blue) present in lipid extracts was quantified by liquid chromatography-mass spectrometry. n=3 and n=2 independent experiments for panels **a** and **b**, respectively, and error bars represent standard deviation from the mean.

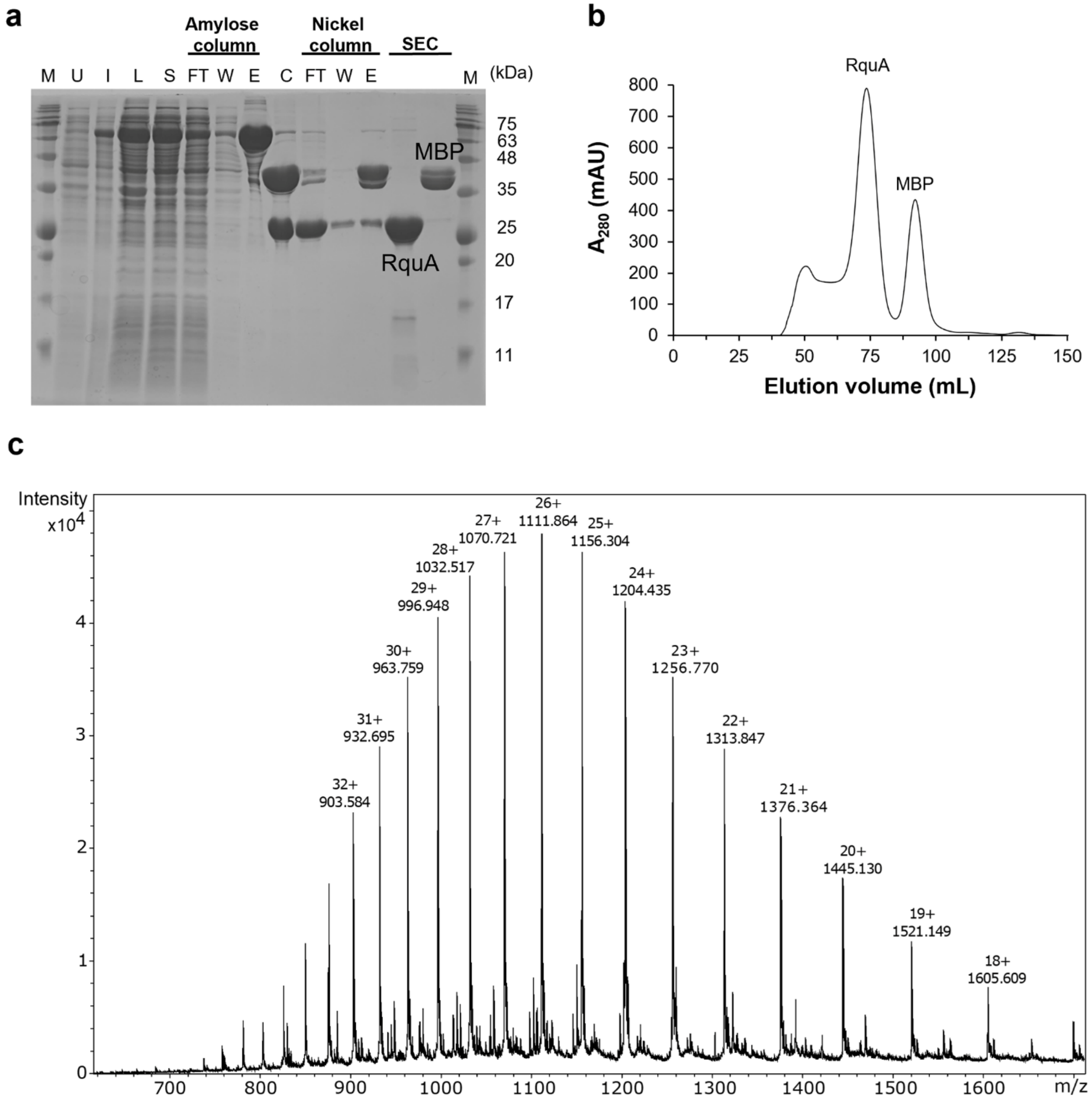

Supplementary Figure 2. Expression and purification of RquA. a SDS-PAGE analysis of RquA purification. After overexpression of pET21_MBP-RquA in *E. coli* XJb (DE3), cells were lysed and maltose binding protein (MBP)-RquA was purified by amylose affinity chromatography, protease cleavage to remove the MBP affinity tag, Ni^{2+} affinity, and size exclusion chromatography (SEC). The identities of purified protein bands are indicated. Gel lanes are U: uninduced cells; I: induced cells; L: cell lysate; S: lysate supernatant; FT: flow-through; W: wash; E: elution; C: after protease cleavage; M: Molecular weight marker. **b** Size-exclusion chromatogram of the nickel column FT fraction. The identities of major peaks are indicated. A calibration curve is presented as Supplementary Fig. 15. **c** Electrospray ionization mass spectrometry analysis of purified RquA. The measured deconvoluted mass of RquA is 28881.48 Da, while the calculated molecular mass of RquA is 28882.97 Da.

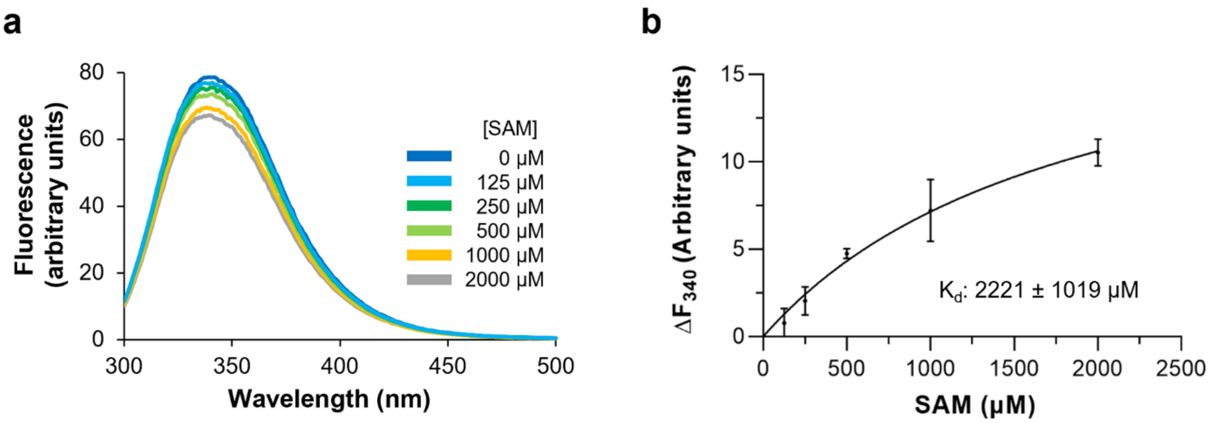

Supplementary Figure 3. Fluorescence spectroscopy-based binding assay. a Emission spectra of RquA were measured from 300 – 500 nm with increasing concentrations of *S*-adenosyl-*L*-methionine (SAM) (dark blue: 0 µM, light blue: 125 µM, dark green: 250 µM, light green: 500 µM, orange: 1000 µM, grey: 2000 µM). **b** A plot of relative change in fluorescence intensity at 340 nm (ΔF_{340}). Data were fitted to a one-site binding model, and the measured dissociation constants (K_d) are indicated. For **b,** n=3 independent experiments and error bars represent the standard deviation from the mean.

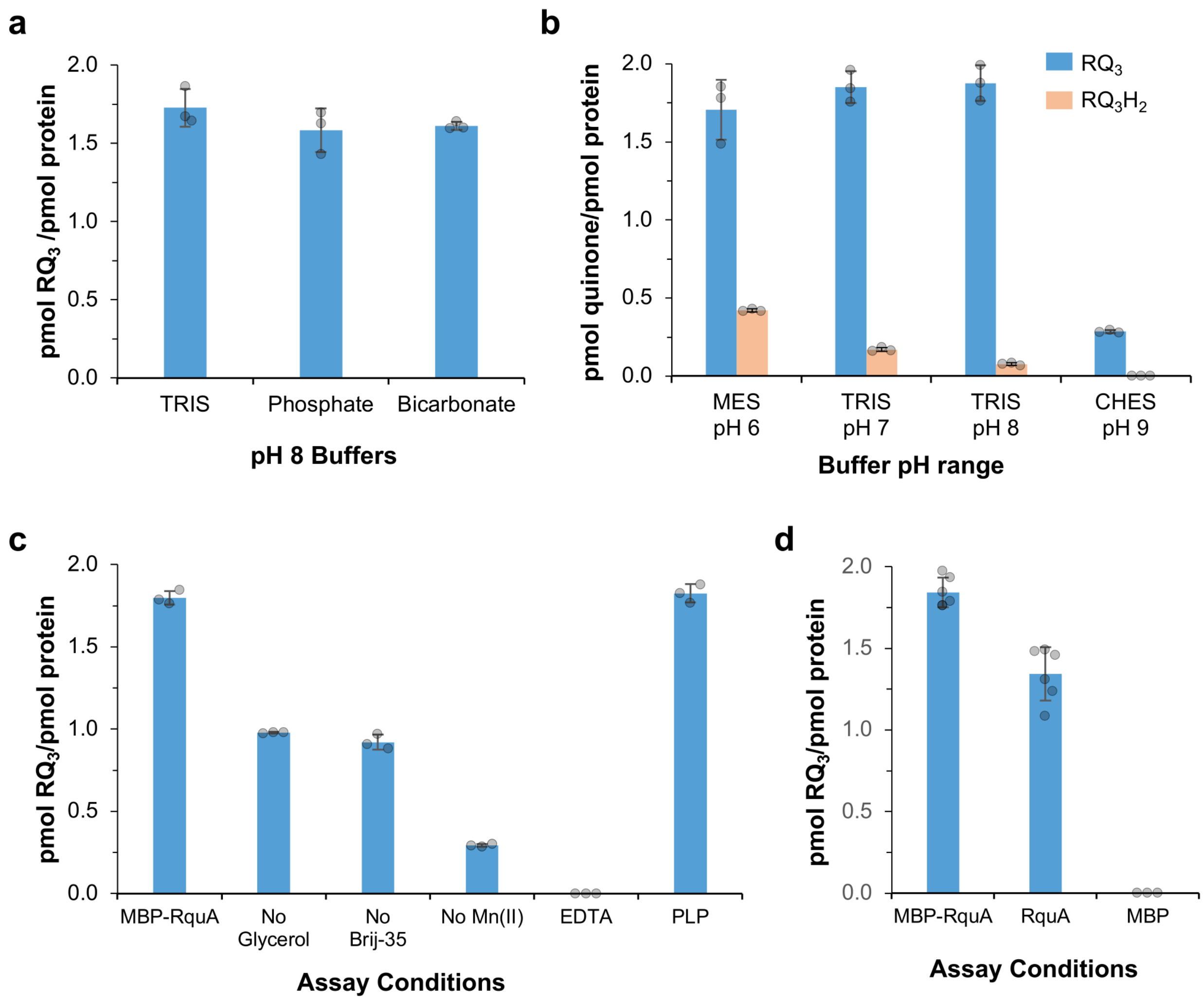

Supplementary Figure 4. MBP-RquA *in vitro* assay optimization. a Three different pH 8 buffers (35 mM TRIS, K_2HPO_4 or $NaHCO_3$) gave similar yields of rhodoquinone-3 (RQ_3) using MBP-RquA (0.5 µM), *S*-adenosyl-*L*-methionine (SAM, 5 µM) and ubiquinone-3 (UQ_3, 1 µM) after 32 min. **b** RQ_3 (blue) and rhodoquinol-3 (RQ_3H_2, orange) production from the same protein and substrate concentrations above was measured at four different pH values using 35 mM concentrations of 2-(*N*-morpholino)ethanesulfonic acid (MES, pH 6), tris(hydroxymethyl)aminomethane (TRIS, pH 7 and 8) or *N*-Cyclohexyl-2-aminoethanesulfonic acid (CHES, pH 9). All assays in panels **a** and **b** contained NaCl (100 mM), tris(2-carboxyethyl) phosphine (TCEP, 0.5 mM), $MnCl_2$ (0.5 mM), Brij-35 (0.05%) and glycerol (10%). **c** RQ_3 yield from a control containing the same protein and substrate concentrations and the complete TRIS assay buffer described above was compared to assays minus glycerol, Brij-35 or Mn^{2+}. These deletions significantly decreased the yield of RQ_3 ($p < 0.001$, n = 3). It was assumed that some Mn^{2+} remained bound to MBP-RquA during the native purification since addition of ethylenediaminetetraacetic acid (EDTA) eliminated all activity. Supplementation of the assay with pyridoxal phosphate (PLP, 1 µM) had no effect on RQ_3 levels. **d** MBP-RquA was cleaved using tobacco etch virus protease and *in vitro* assays were performed using the complete TRIS buffer conditions above with maltose binding protein (MBP)-RquA, cleaved RquA, or MBP. RQ_3 is produced from RquA with and without the MBP tag. n=3 independent experiments and error bars represent standard deviation from the mean.

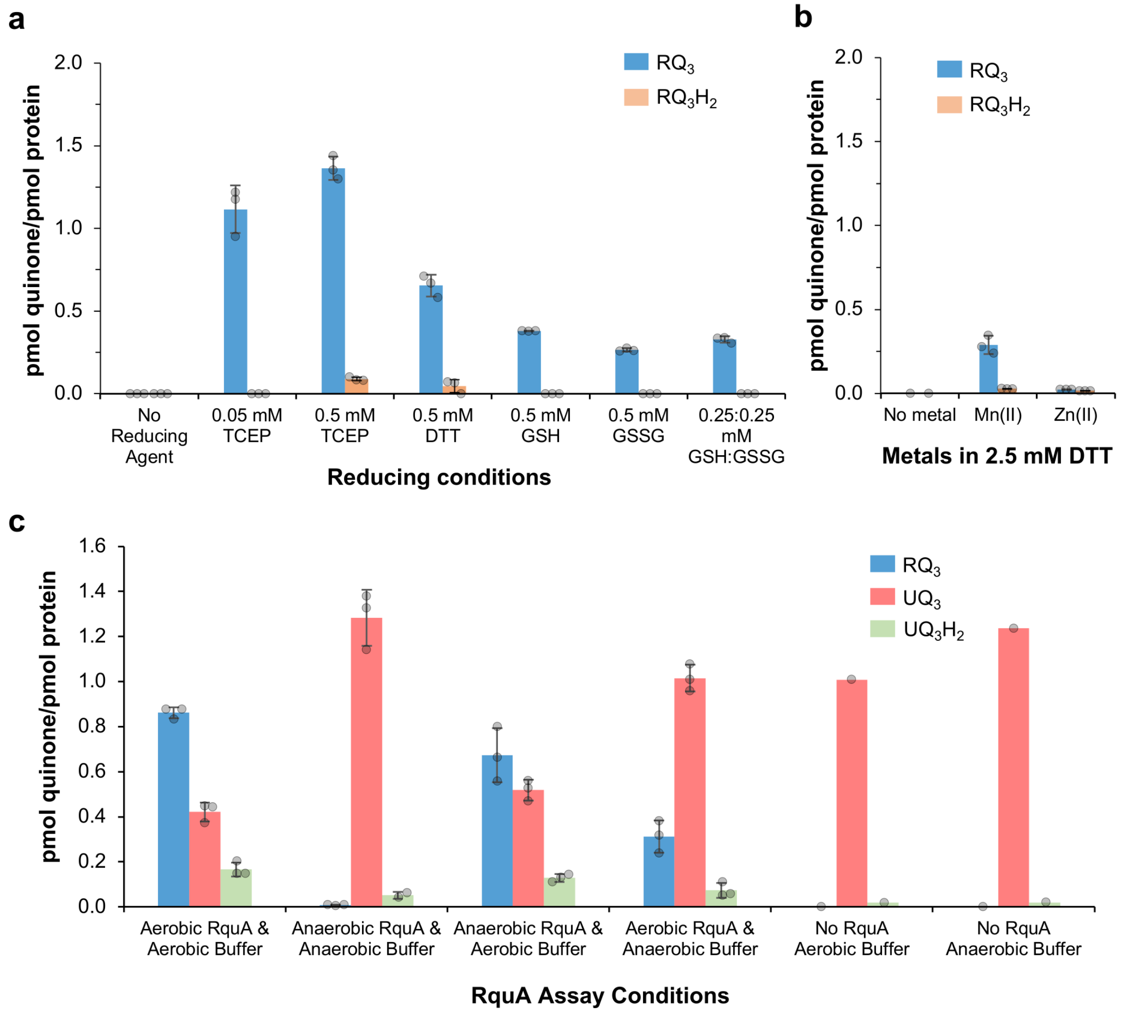

Supplementary Figure 5. RQ_3 produced during *in vitro* assays with cleaved RquA or MBP-RquA using different reducing agents. a RquA was dialyzed in buffer containing tris(2-carboxyethyl) phosphine (TCEP), dithiothreitol (DTT), reduced glutathione (GSH) or no reducing agent. The dialyzed proteins were diluted (0.5 µM final) in buffer supplemented with $MnCl_2$ (0.5 mM final) and additional TCEP, DTT, GSH and/or oxidized glutathione (GSSG) was added to the final concentrations listed. All assays contained *S*-adenosyl-*L*-methionine (SAM, 5 µM) and were initiated by addition of ubiquinone-3 (UQ_3, 1 µM). **b** MBP-RquA was diluted to 0.5 µM in buffer containing 2.5 mM DTT and treated with ethylenediaminetetraacetic acid (EDTA, 0.5 mM) for 5 min before addition of either Mn^{2+} or Zn^{2+} (1 mM $MnCl_2$ or $ZnSO_4$). SAM and UQ_3 were added to assays at same concentrations as above. Assays with Zn^{2+} generated small amounts of rhodoquinone-3 (RQ_3) and rhodoquinol-3 (RQ_3H_2) only under high concentrations of DTT (and under none of the conditions listed in panel **a**). **c** Assays were performed with RquA using the same concentrations of SAM and UQ_3 as above and a tris(hydroxymethyl)aminomethane (TRIS) buffer containing 0.05 mM TCEP under varying levels of oxygen: i. aerobically (in air); ii. Anaerobically (under argon); iii. Anaerobic RquA (sparged with argon) mixed with aerobic buffer; and iv. Aerobic RquA mixed with anaerobic buffer (sparged with argon). No RQ_3 was produced in the complete absence of oxygen or protein. When present, error bars represent standard deviation from the mean of n=3 independent experiments. Production of RQ_3, RQ_3H_2, UQ_3, and ubiquinol-3 (UQ_3H_2) are shown as blue, orange, red, and green bars, respectively.

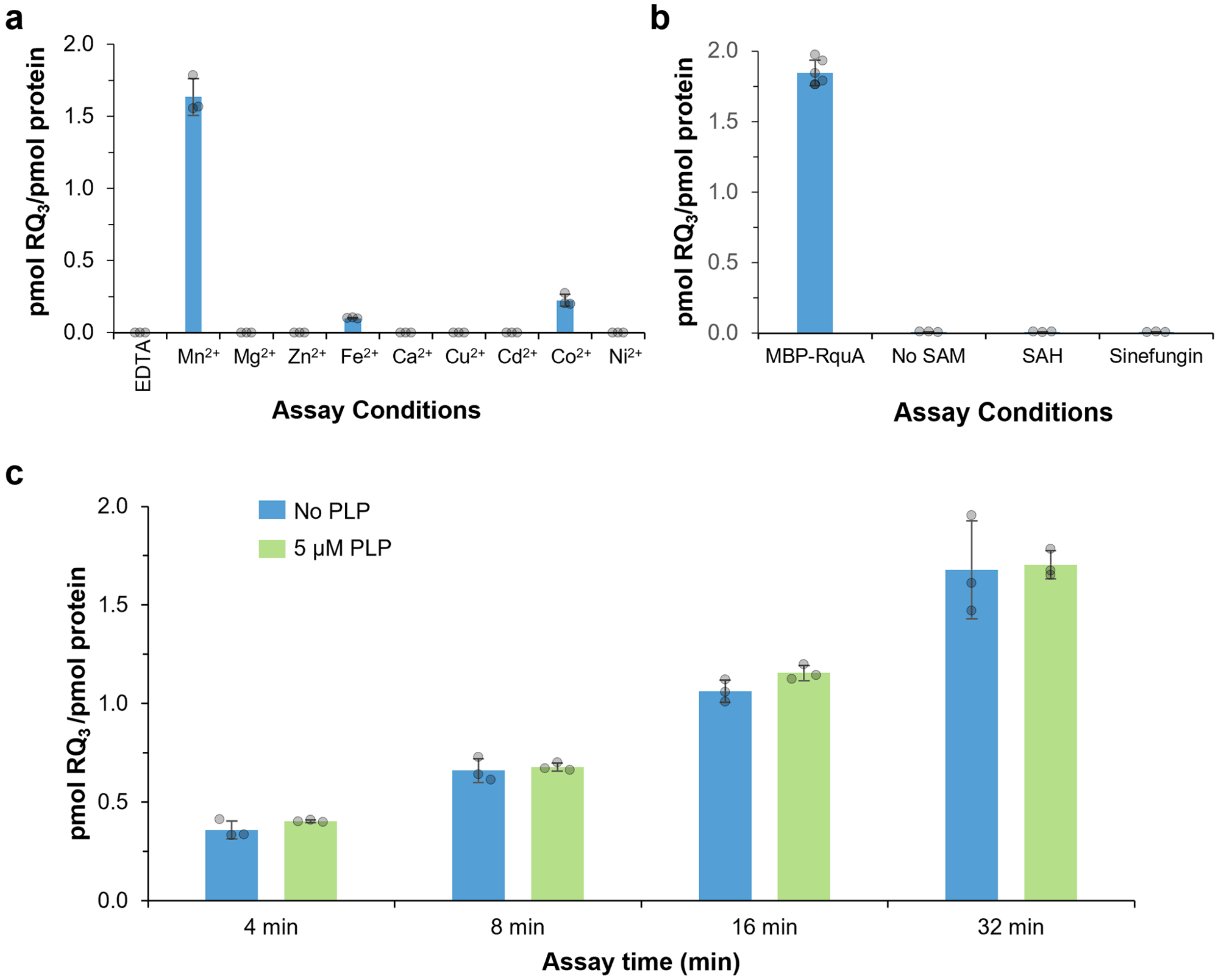

Supplementary Figure 6. Metal cofactors and PLP in RQ biosynthesis. a Metal rescue assays were completed using assay buffer containing tris(hydroxymethyl)aminomethane (TRIS, 35 mM), NaCl (100 mM), $MnCl_2$ (0.5 mM), Brij-35 (0.05%) and glycerol (10%) adjusted to pH 8. MBP-RquA (0.5 µM) was first treated with ethylenediaminetetraacetic acid (EDTA, 0.5 mM), followed by addition of nine different M^{2+} solutions (1 mM) before *S*-adenosyl-*L*-methionine (SAM, 5 µM) and ubiquinone-3 (UQ_3, 1 µM) substrates were added. Reactions were allowed to proceed for 32 min at rt. **b** An MBP-RquA control was performed with SAM and compared to assays containing no SAM, or with SAM replaced by *S*-adenosyl-*L*-homocysteine (SAH) or sinefungin. **c** Assays with cleaved RquA (0.5 µM), SAM (5 µM) and UQ_3 (1 µM) were performed with (green) and without (blue) pyridoxal phosphate (PLP, 5 µM) in the assay buffer described above. Assays were performed at rt and quenched with HCl at four different time points. There was no significant difference in RQ_3 production with the addition of PLP. n=3 independent experiments and error bars represent standard deviation from the mean.

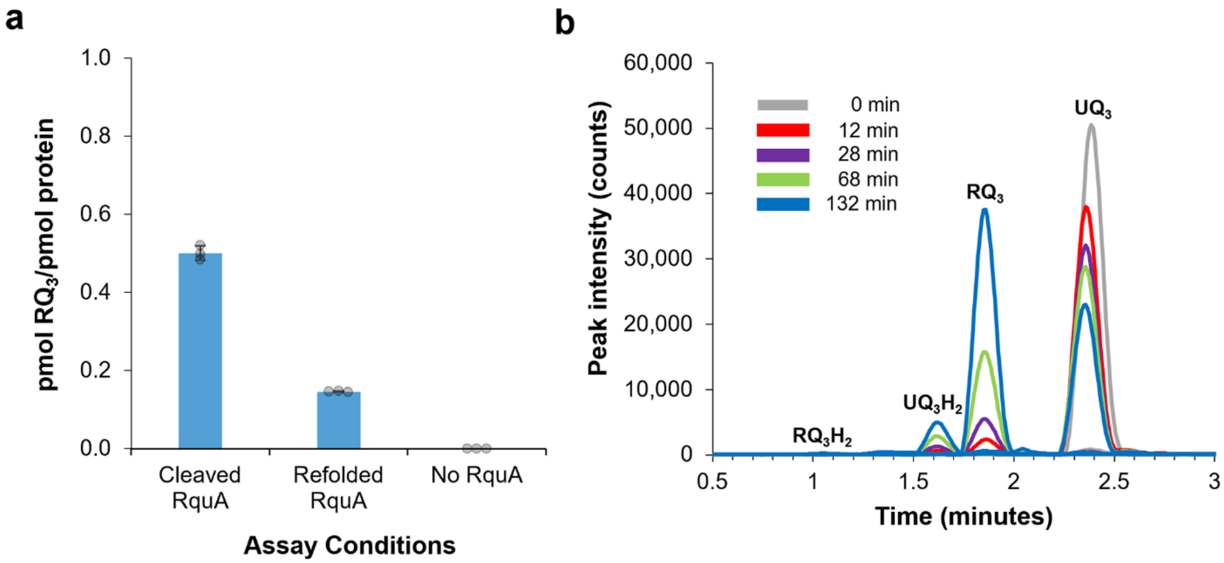

Supplementary Figure 7. Quinones produced from MBP-cleaved or refolded RquA. RquA used for refolding was expressed in XJb (DE3) *E. coli* using the pET303_RquA vector and purified by nickel affinity chromatography from inclusion bodies denatured in 8 M urea. Refolding was performed by dialysis in buffer containing tris(hydroxymethyl)aminomethane (TRIS, 20 mM), NaCl (200 mM), tris(2-carboxyethyl) phosphine (TCEP, 1 mM), $MnCl_2$ (0.5 mM), 0.1% Brij-35 and 10% glycerol at pH 8. **a** *In vitro* assays quantifying rhodoquinone-3 (RQ_3), rhodoquinol-3 (RQ_3H_2), ubiquinone-3 (UQ_3), and ubiquinol-3 (UQ_3H_2) were performed as described above with or without RquA (1 μM). n=3 independent experiments and error bars represent standard deviation from the mean. **b** Time course chromatograms (grey: 0 min, red: 12 min, purple: 28 min, green: 68 min, blue: 132 min) were collected for an *in vitro* assay with refolded RquA (1 μM) after UQ_3 (1 μM) was added.

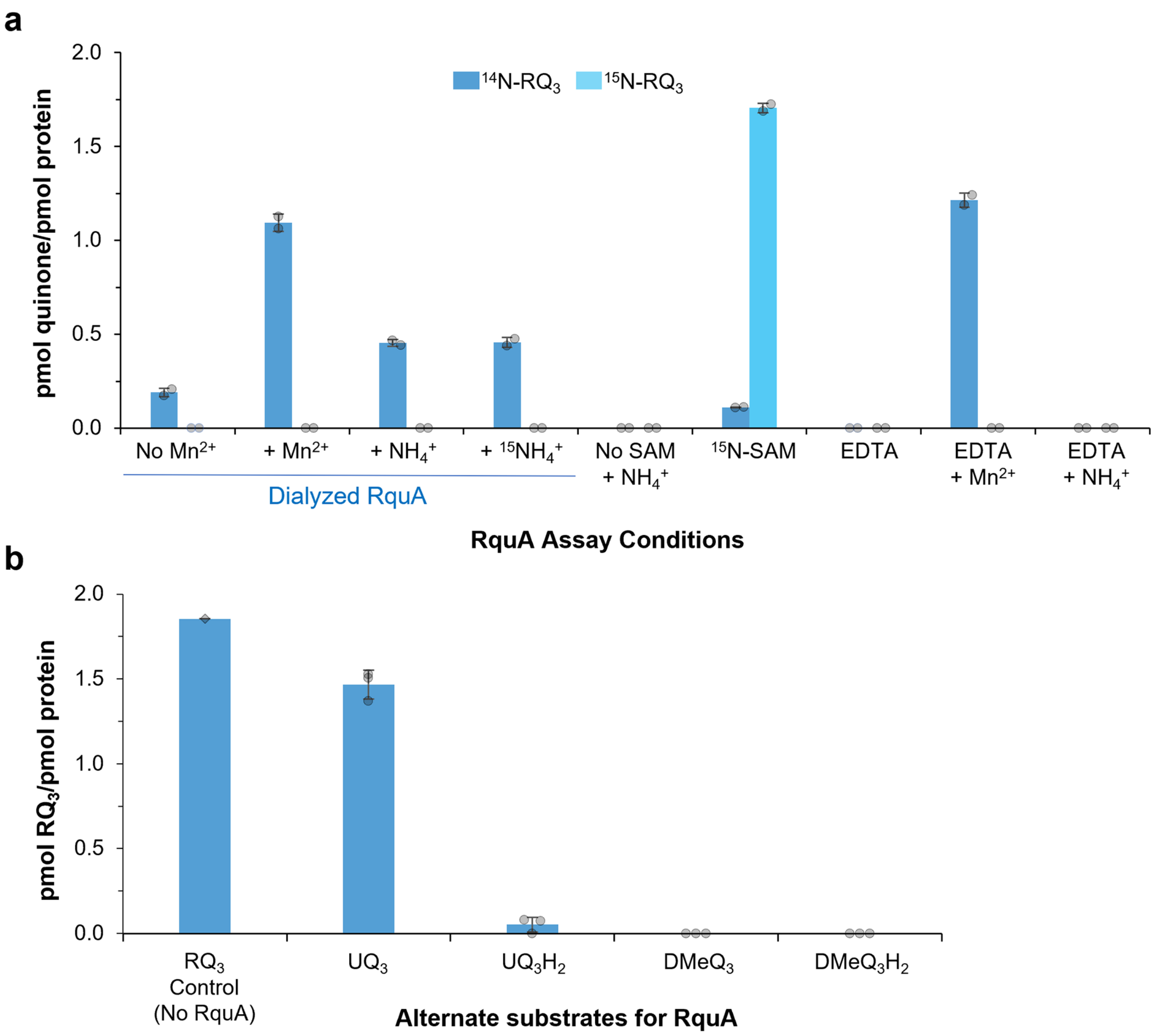

Supplementary Figure 8. Alternate nitrogen sources and substrates for RquA assays. a RquA (1 µM) was dialyzed overnight to remove free Mn^{2+}. Assays for ^{14}N-rhodoquinone-3 (^{14}N-RQ_3, dark blue) and ^{15}N-RQ_3 (light blue) were performed using ubiquinone-3 (UQ_3, 1 µM) and *S*-adenosyl-*L*-methionine (SAM, 5 µM) with tris(hydroxymethyl)aminomethane (TRIS) pH 8 buffer containing tris(2-carboxyethyl) phosphine (TCEP, 0.5 mM), Brij-35 (0.05%), and glycerol (10%) for 64 min. Adding supplemental Mn^{2+} restored activity and NH_4^+ also increased activity compared to the No Mn^{2+} control. Supplementation with $^{15}NH_4Cl$ did not produce ^{15}N-RQ_3 and NH_4^+ could not replace SAM as the amino source; however, substitution of ^{14}N-SAM with ^{15}N-SAM generated ^{15}N-RQ_3 (from Fig. 4 in text). After ethylenediaminetetraacetic acid (EDTA) treatment of RquA, only Mn^{2+} could restore activity. n=2 independent experiments and error bars represent standard deviation from the mean. **b** *In vitro* assays were performed using cleaved RquA (0.5 µM), SAM (5 µM) and UQ_3, ubiquinol-3 (UQ_3H_2), demethylubiquinone-3 ($DMeQ_3$) or demethylubiquinol-3 ($DMeQ_3H_2$) substrates (1 µM) and monitored for rhodoquinone-3 (RQ_3) production after 32 min using the assay conditions described previously. A no protein control with RQ_3 (1 µM) is shown for reference. RQ_3 produced from UQ_3H_2 was likely due to air oxidation of the substrate to UQ_3 during the assay (the oxidation product then likely reacted with RquA to form a small amount of RQ_3). n=3 independent experiments and error bars represent standard deviation from the mean.

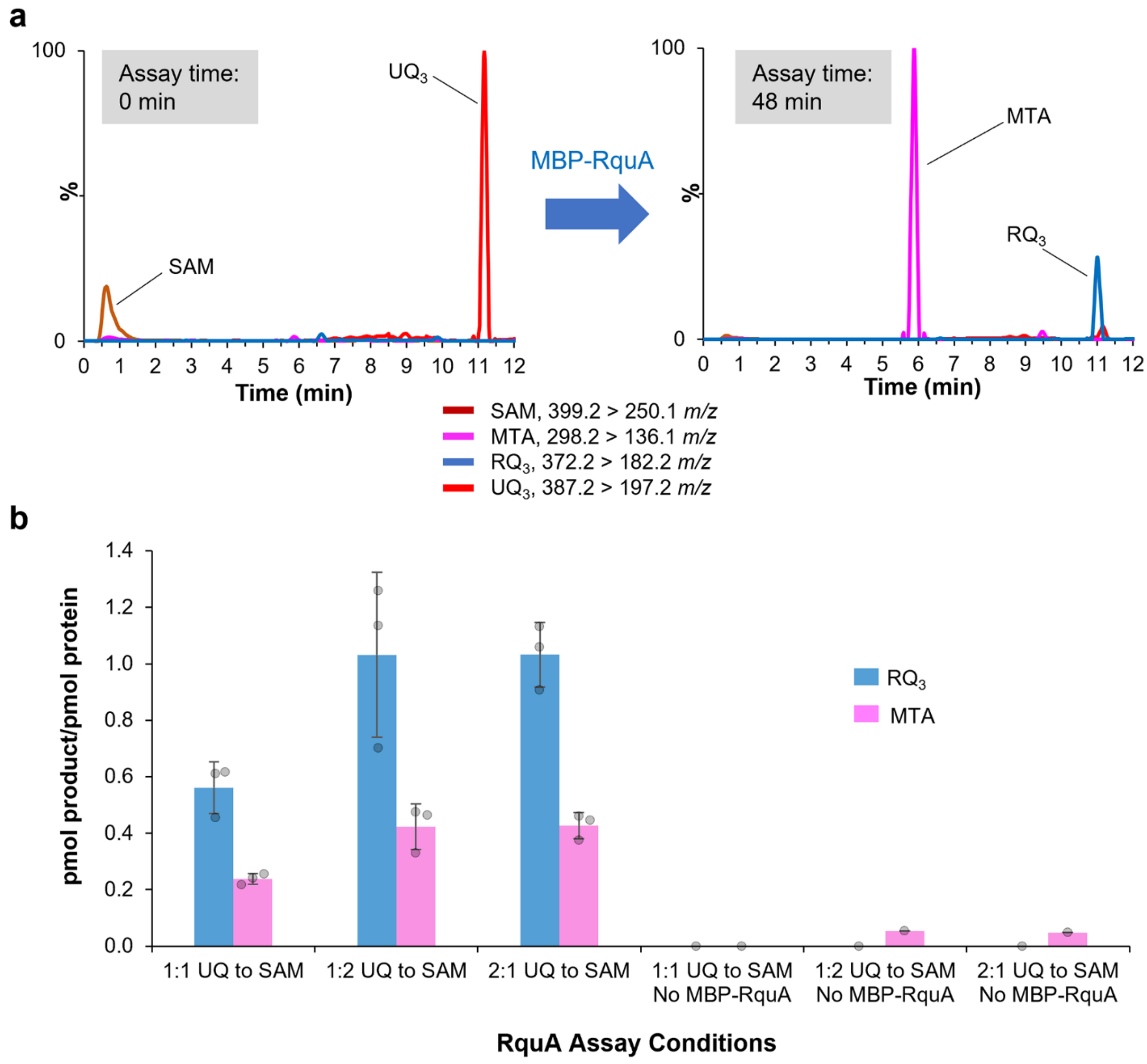

Supplementary Figure 9. MTA is a product generated from SAM during the RquA *in vitro* assay. a Assays were performed using purified *S*-adenosyl-*L*-methionine (SAM, 5 µM), ubiquinone-3 (UQ_3, 5 µM) and MBP-RquA (0.5 µM) in 2-(*N*-morpholino)ethanesulfonic acid (MES) assay buffer adjusted to pH 6 and sampled after 48 min at rt. Mass chromatograms from before (left panel) and 48 min after (right panel) addition of MBP-RquA are overlaid. Four multiple reaction monitoring transitions were monitored during the ^{14}N-SAM assay: 399.2 > 250.1 *m/z* (^{14}N-SAM, maroon), 298.2 > 136.1 *m/z* (^{12}C-methyl methylthioadenosine (MTA), magenta), 372.2 > 182.2 *m/z* (^{14}N-rhodoquinone-3 (^{14}N-RQ_3), blue) and 387.2 > 197.2 *m/z* (UQ_3, red). The response factor for MTA is approximately four times larger than for RQ_3 and chromatogram peak areas cannot be compared directly. **b** Substrate stoichiometry was varied between 1-2 µM to achieve molar ratios of 1:1, 1:2, and 2:1 UQ_3:SAM. RQ_3 (blue) and MTA (magenta) were quantified in the presence and absence of MBP-RquA (0.5 µM). The amount of MTA increased significantly with the formation of RQ_3 (versus SAM decomposition to MTA in the absence of protein). Doubling the amount of UQ_3 or SAM had a similar increase in the amount of MTA and RQ_3. N=3 independent experiments and error bars represent the standard deviation from the mean. No protein controls were performed in single sets.

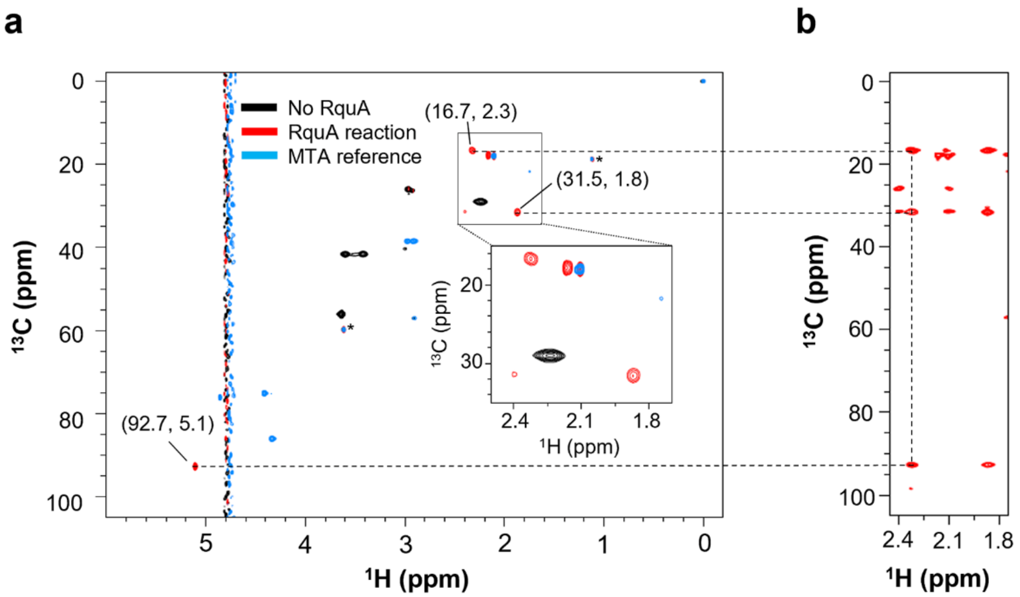

Supplementary Figure 10. NMR-based identification of MTA and an aldehyde hydrate as RquA reaction products. a Overlay of ^{1}H-^{13}C HSQC spectra of an *in-vitro* assay performed using ubiquinone-3 (UQ_3, 350 µM) and $^{13}C_5{}^{15}N$-*S*-adenosyl-*L*-methionine (SAM, 200 µM). Spectra are shown for the *in vitro* assay lacking RquA (black), the *in vitro* assay after addition of RquA (red), and for an methylthioadenosine (MTA) reference in assay buffer (blue). Peaks corresponding to the aldehyde hydrate product have their chemical shifts indicated as ($\delta^{13}C$, $\delta^{1}H$). The inset shows an overlay of the reaction products with the CH_3 resonance of MTA. Signals arising from buffer components are indicated with *. **b** A selected region of the HSQC-TOCSY spectrum that shows correlations between the resonances indicated in panel **a**.

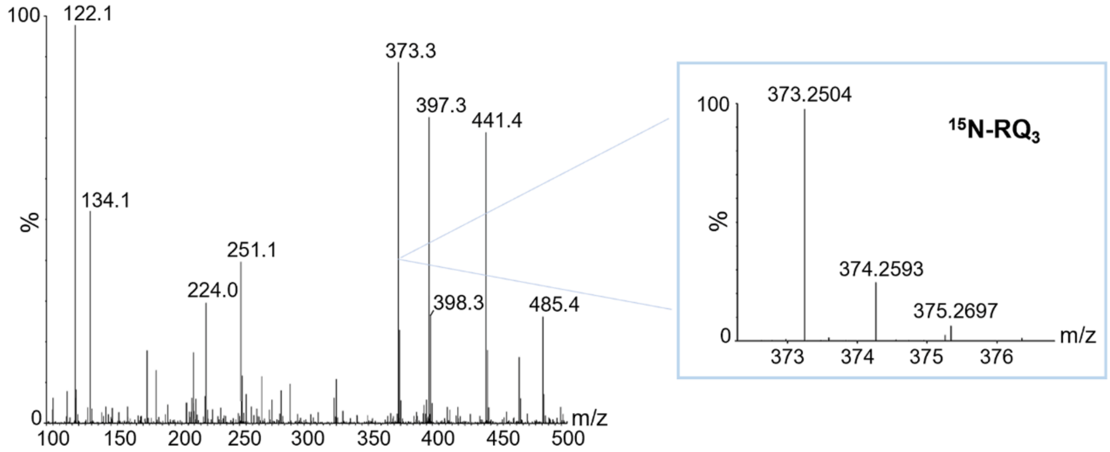

Supplementary Figure 11. Full mass spectrum of ^{15}N-rhodoquinone (RQ$_3$). The mass of synthesized [^{15}N-RQ$_3$ + H]$^+$ was determined to be 373.2504 *m/z* ($C_{23}H_{34}{}^{15}NO_3$ exact mass = 373.2509 *m/z*, mass accuracy = -1.4 ppm).

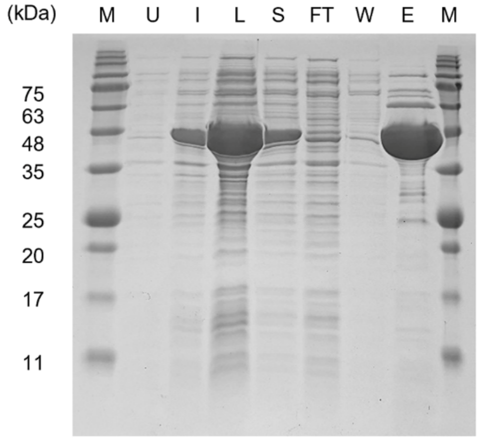

Supplementary Figure 12. Expression and purification of *S*-adenosyl-*L*-methionine (SAM) synthetase. pET21_SAMS was transformed into *E. coli* BL21 (DE3) cells. After overexpression collected cells were lysed and purified by nickel affinity chromatography. Gel lanes are U: uninduced cells; I: induced cells; L: cell lysate; S: lysate supernatant; FT: flow-through; W: wash; E: elution; M: molecular weight marker.

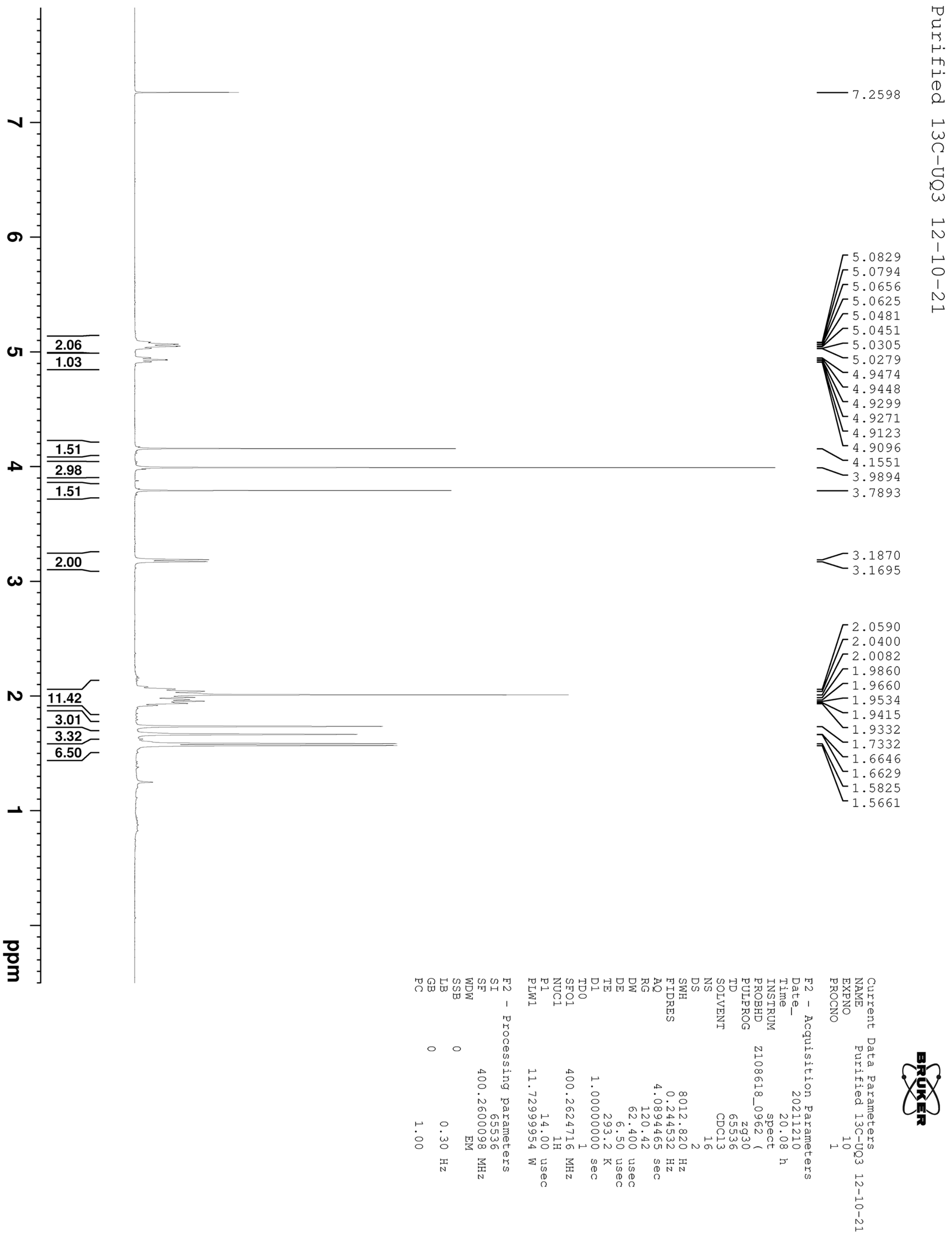

Supplementary Figure 13. ^{1}H NMR spectrum of 5-^{13}C-methoxy-ubiquinone-3. A 400 MHz ^{1}H NMR spectrum of synthesized 5-^{13}C-methoxy-ubiquinone-3 (UQ_3) measured in $CDCl_3$. Chemical shifts and integrals of picked peaks are indicated. A doublet (3.79 ppm and 4.16 ppm) is observed due to the incorporation of ^{13}C for one of the methoxy groups of UQ_3.

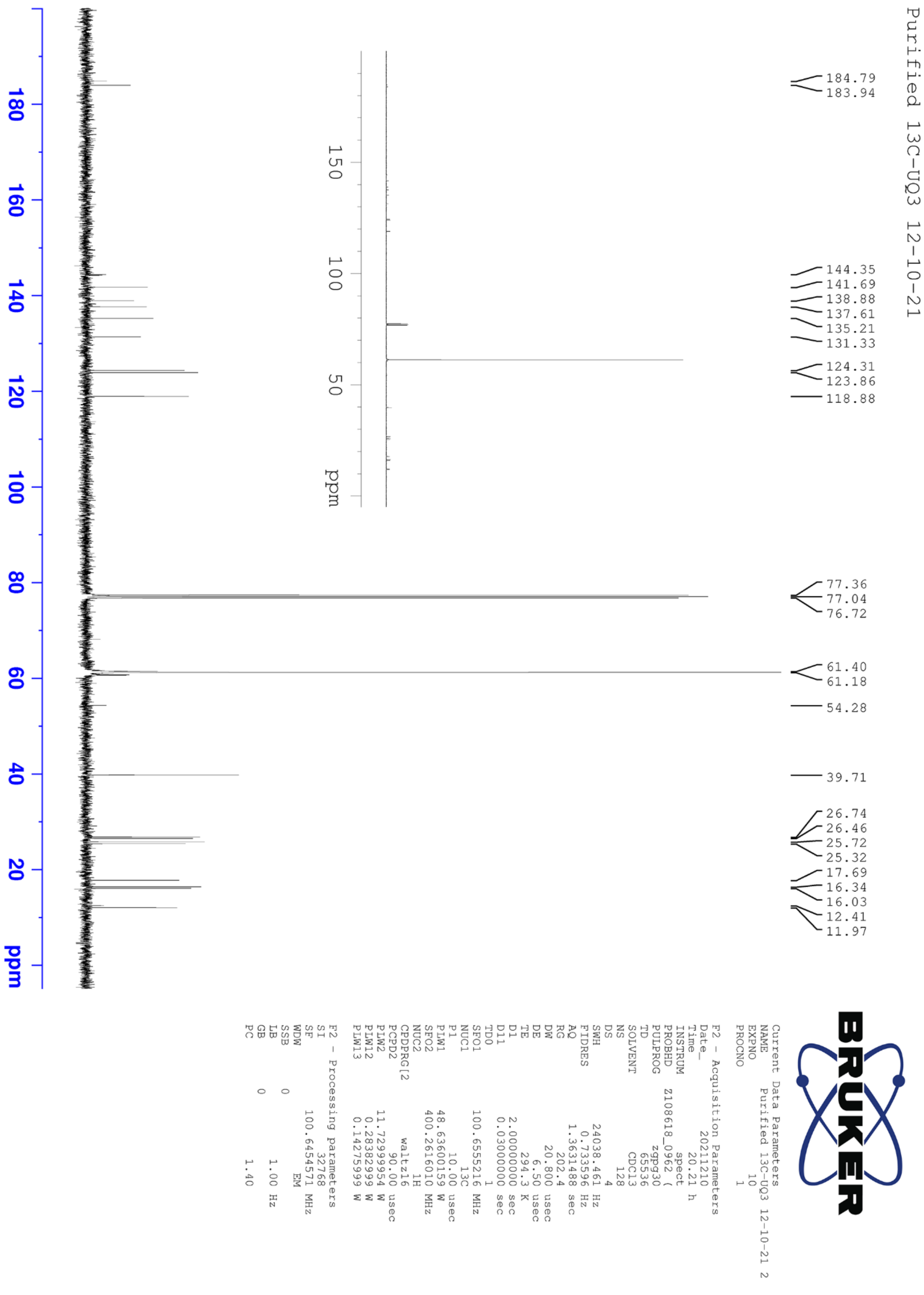

Supplementary Figure 14. ^{1}H-decoupled ^{13}C NMR spectrum of 5-^{13}C-methoxy-ubiquinone-3. A 400 MHz ^{1}H-decoupled ^{13}C NMR spectrum of synthesized 5-^{13}C-methoxy-ubiquinone-3 (UQ_3) measured in $CDCl_3$. Chemical shifts are indicated. Due to ^{13}C-enrichment of the 5-methoxy group the intensity of the peak at ~61.4 ppm is cut off, and its true intensity is indicated with the inset.

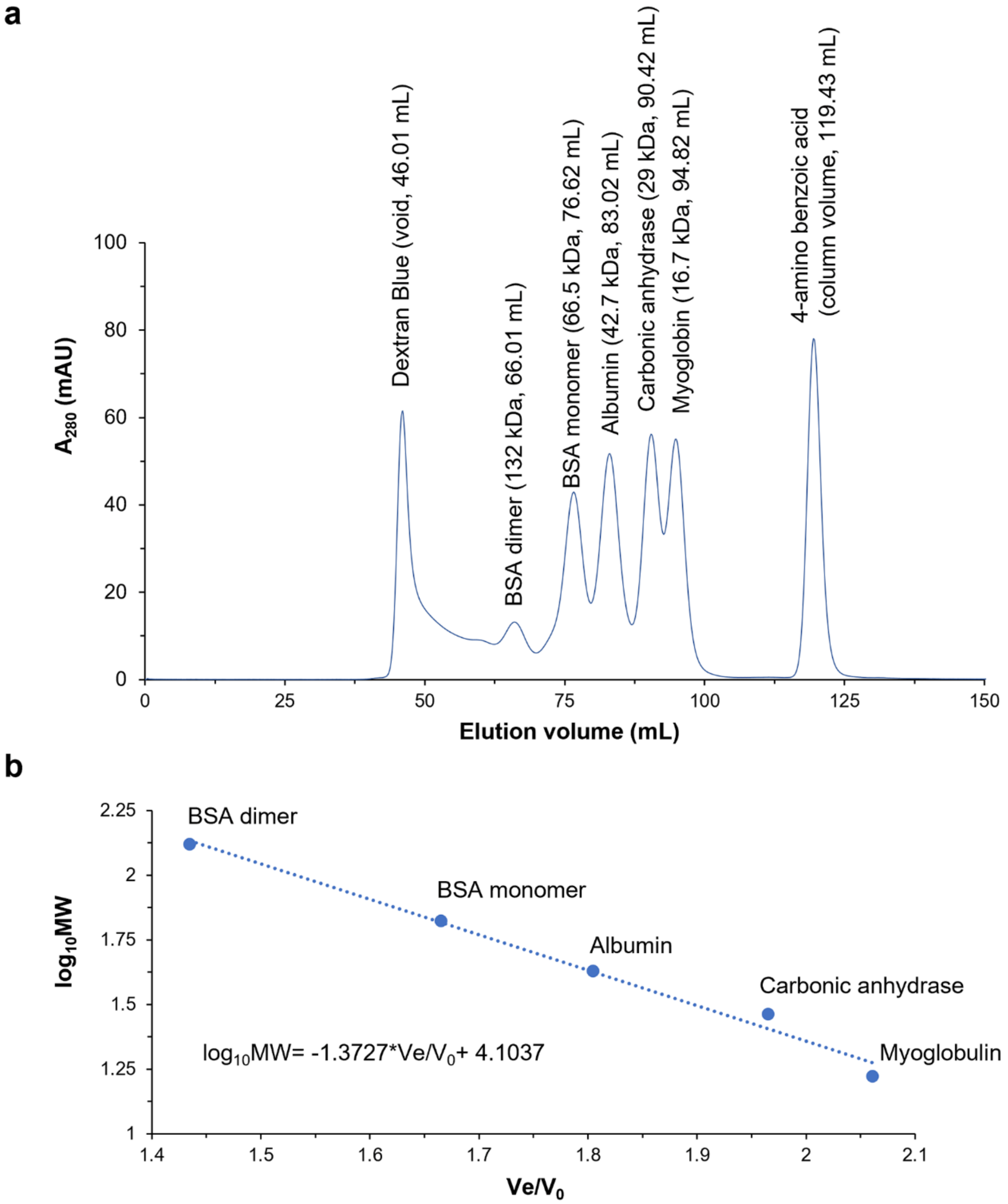

Supplementary Figure 15. Calibration of the preparative size exclusion chromatography column (HiLoad 16/60) using the proteins of known molecular size. **a** Elution profile of the protein standards with their size and elution volume in parenthesis. **b** Molecular weight (MW) calibration curve for protein standards. RquA elutes at 73.66 mL, which corresponds to 80.6 kDa based on the calibration curve equation where Ve and V_0 represent the elution volume of each protein and the void volume (46.01 mL) of the column, respectively.

Supplementary Table 1. Oligonucleotides used in this study. Restriction endonuclease sites are underlined in the sequence.

Name	Direction	Restriction site	Sequence (5’-3’)
P1	Forward	EcoRI	CAGTGAATTCGATGACTAAGCACCAAGGTG
P2	Reverse	BamHI	ACGTGGATCCTTAAGCGCGTCGCTC
P3	Forward	EcoRI	GGCCGCGAATTCAGCGTATTTGCAAGAGACCT
P4	Reverse	BamHI	ATTATTGGATCCTTAAGCGCGTCGCTCCGCGACG
P5	Forward	BglII	CGAAGCAGATCTGCAAAACACCTTTTTACGTCC
P6	Reverse	XhoI	CGAAGCCTCGAGTTACTTCAGACCGGCAGCATC

Supplementary Table 2. Liquid chromatography-mass spectrometry parameters for detection of analytes using C-18 column (15 min method)

MS parameter	^{14}N-SAM	^{13}C-Me SAM	$^{13}C_5{}^{15}$N-SAM	MTA	^{13}C-Me MTA	UQ_1
[1]Dwell time (s)	0.1	0.1	0.1	0.1	0.1	0.1
Cone (V)	20	20	20	25	25	18
Collision (V)	17	17	17	16	16	12
Precursor mass $[M+H]^+$ (*m*/*z*)	399.2	400.2	405.2	298.2	299.2	251.1
Ion product mass $[M]^+$ (*m*/*z*)	250.1	250.1	250.1	136.1	136.1	219.1

[1]Dwell times of 0.025 s were used on the Waters Xevo TQ-S Cronos

Supplementary Table 3. Liquid chromatography-mass spectrometry parameters for detection of quinones using PFP column (6.5 min method)

MS parameter	^{15}N-RQ$_3$	RQ$_3$H$_2$	UQ$_3$H$_2$	UQ$_4$	^{15}N-RQ$_6$
[1]Dwell time (s)	0.1	0.1	0.1	0.1	0.1
Cone (V)	25	25	20	24	35
Collision (V)	20	20	20	23	28
Precursor mass $[M+H]^+$ (*m*/*z*)	373.2	374.2	389.2	455.3	577.4
Ion product mass $[M]^+$ (*m*/*z*)	183.2	182.2	197.2	197.2	183.2

[1]Dwell times of 0.025 s were used on the Waters Xevo TQ-S Cronos
